# Genetic dissection of root architectural plasticity and identification of candidate loci in response to drought stress in bread wheat

**DOI:** 10.1186/s12863-023-01140-7

**Published:** 2023-07-26

**Authors:** Nurealam Siddiqui, Melesech T. Gabi, Mohammad Kamruzzaman, Abebaw M. Ambaw, Tesfaye J. Teferi, Said Dadshani, Jens Léon, Agim Ballvora

**Affiliations:** 1 Institute of Crop Science and Resource Conservation (INRES)-Plant Breeding, University of Bonn, 53115 Bonn, Germany; 2 Department of Biochemistry and Molecular Biology, Bangabandhu Sheikh Mujibur Rahman Agricultural University, Gazipur, 1706 Bangladesh; 3 Plant Breeding Division, Bangladesh Institute of Nuclear Agriculture (BINA), Mymensingh-2202, Bangladesh; 4 INRES-Plant Nutrition, University of Bonn, 53115 Bonn, Germany; 5 Field Lab Campus Klein-Altendorf, University of Bonn, Klein-Altendorf 2, 53359 Rheinbach, Germany

**Keywords:** Candidate loci, Drought stress, GWAS, Root phenotypic plasticity, SNP, Wheat

## Abstract

**Background:**

The frequency of droughts has dramatically increased over the last 50 years, causing yield declines in cereals, including wheat. Crop varieties with efficient root systems show great potential for plant adaptation to drought stress, however; genetic control of root systems in wheat under field conditions is not yet well understood.

**Results:**

Natural variation in root architecture plasticity (phenotypic alteration due to changing environments) was dissected under field-based control (well-irrigated) and drought (rain-out shelter) conditions by a genome-wide association study using 200 diverse wheat cultivars. Our results revealed root architecture and plasticity traits were differentially responded to drought stress. A total of 25 marker-trait associations (MTAs) underlying natural variations in root architectural plasticity were identified in response to drought stress. They were abundantly distributed on chromosomes 1 A, 1B, 2 A, 2B, 3 A, 3B, 4B, 5 A, 5D, 7 A and 7B of the wheat genome. Gene ontology annotation showed that many candidate genes associated with plasticity were involved in water-transport and water channel activity, cellular response to water deprivation, scavenging reactive oxygen species, root growth and development and hormone-activated signaling pathway-transmembrane transport, indicating their response to drought stress. Further, *in silico* transcript abundance analysis demonstrated that root plasticity-associated candidate genes were highly expressed in roots across different root growth stages and under drought treatments.

**Conclusion:**

Our results suggest that root phenotypic plasticity is highly quantitative, and the corresponding loci are associated with drought stress that may provide novel ways to enable root trait breeding.

**Supplementary Information:**

The online version contains supplementary material available at 10.1186/s12863-023-01140-7.

## Introduction

Globally, 90–95% of the produced wheat is the hexaploid common wheat (*Triticum aestivum* L.), an important stable source of nutrients and fodder for the majority of the world’s population [1, 2]. As the global population is rapidly growing, the demand for wheat will also be high; thus, wheat production needs to increase to 70% by 2050 [3]. Although the demand for wheat is becoming high, its production is being constrained by various abiotic factors, with drought being the main factor reducing wheat production by approximately 20% [4], including the following main climatic factors: water scarcity, flooding, high and low-temperature stress, making wheat vulnerable to yield losses during the grain-filling period.

Drought stress is the absence or lack of water in a given environment that could alter the biochemical, physiological and molecular systems of a plant. It is the main factor affecting the broad spectrum of agro-climatic production and productivity of wheat [2]. Several biological processes regulate the drought tolerance of plants, which in turn affects grain yield [5]. The root system architecture is a major factor for plant adaptation under different climatic conditions, including water stress conditions [6, 7]. Wheat is categorised as a monocot root system possessing both seminal and adventurous roots [8]. There are several root morphological traits, which provide structural and mechanical strength of root system architecture in wheat contributing to water-deficit adaptation [7, 9]. The root traits of wheat, particularly during water scarcity, are important for water absorption and are also essential for nutrient uptake, such as nitrogen and phosphorus [10]. During water-deficit stress, plants tend to change their root architectural structure, e.g. branched roots and increased root length, to meet their water needs [11]. Therefore, rooting depth is considered an essential trait that may enhance the plant’s ability to minimise reduced productivity, especially when insufficient soil moisture is available. In deep soils with water reserves, a deep root system is crucial in drought tolerance [7, 12]. Root architectural traits that help improve the water uptake during drought stress conditions are proliferative rooting, such as lateral root number, length density, surface area and volume [13]. The plant also adapts to drought stress by modulating the root system traits, decreasing lateral root diameter, and altering its root biomass [14]. Moreover, cereal roots show plasticity to adapt to drought. Plasticity is caused by the phenotypic changes when exposed to variable environments may be of short or long duration. Plasticity in root phenotypes can be beneficial for drought adaptation [15, 16]. Recently, the genetic basis of root plasticity in enhancing drought adaptation has been successfully uncovered by a genome-wide association study (GWAS) [17, 18].

In the past few decades, the grain yield and quality of bread wheat have greatly focused on breeding. Due to population growth and climate changes, wheat adaptation to environmental stress conditions should be further improved. Using state-of-the-art phenotyping and sequencing methods, deep genetic and molecular bases of drought stress tolerance in wheat should be analysed and applied [19, 20]. Although the roots play an important role in plant tolerance to abiotic stresses and productivity, plant breeders mostly focus on above-ground traits because of the difficulty in investing in below-ground traits due to precise phenotyping, especially in large populations [21]. Field-based genetic dissection of the root phenotypic traits in wheat, particularly at the flowering stage, is limited compared to other crops due to its genetic complexity and extensive root phenotyping. Due to limitations of field-based root phenotyping, rapid phenotyping strategies should be utilised to better understand genetic responses and rapid selection of root traits and associated genetic components to develop resilient wheat varieties through molecular breeding, showing the necessity of ensuring future food security [7, 22]. Nowadays, however, a rapid and popular root phenotyping method, known as “Shovelomics” is being used by digging up the upper part of the root systems in the field to phenotype the plant root system and/or root architectural traits [23, 24]. Nevertheless, “Shovelomics” is a well-established and reputed approach for field-based root phenotyping [23], and is reported that traits evaluated in the upper zone (around 30 cm soil depth) are indicative for the traits related to deep rooting [25]. The prominent root traits like root convex area, root surface area (RSA), total root length (TRL) and the root volume (RV) of the upper zone are indicators of the whole root system development [25].

A GWAS applying a diverse association mapping panel with higher allelic diversity and historical recombination has a higher resolution than biparental quantitative trait loci (QTL) studies [26]. High-density single nucleotide polymorphisms (SNPs) are a prerequisite for a successful GWAS [27, 28]. Furthermore, using the drought tolerance index and plasticity in GWAS provides valuable information for marker-assisted selection in wheat [18, 29]. Therefore, GWAS has popularly become an essential tool in identifying SNPs/alleles associated with complex traits, such as root-related traits in bread wheat that provides a genetic basis for identifying causal genes [30].

Given the significance of the investigation of root-mediated bread wheat tolerance to drought stress and its associated genetic architecture, we formulated the following objectives: (i) to assess the root phenotypic diversity of winter bread wheat cultivars grown under control and drought stress conditions, (ii) to identify drought-responsive loci underlying candidate genes associated with root phenotypic plasticity responses and (iii) determine transcript expression levels of plasticity-associated genes in comparing different organs, including roots and drought stress conditions, as they showed involvement in drought.

## Results

### Root phenotypic diversity and correlation analysis in response to drought stress

Root architecture-related traits in wheat cultivars were evaluated under both control and water-deficit stress conditions at the complete flowering stage (BBCH65, Biologische Bundesanstalt, Bundessortenamt und Chemische Industrie) to identify the candidate genes underlying drought-responsive loci by employing a GWAS. A two-way analysis of variance (ANOVA) showed that the effects of genotypes were highly significant (*P* < 0.001) among most of the phenotypic traits, except root average diameter (RAD) showed a weak significant difference (*P* < 0.05) (Table 1). Moreover, treatment effects revealed highly significant differences for all studied traits. Interaction between the genotype and treatment demonstrated highly significant differences among all analysed traits (*P* > 0.05) (Table 1). The heritability calculation demonstrated a high broad-sense heritability (H^2^) for all studied traits, such as TRL, RSA, RAD, number of root tips (NRT), number of root forks (NRF), number of root crossings (NRC) and RV with ranges from 0.73 to 0.87, indicating that the wheat association panel may harbour a wider range of genetic diversity in root traits to confer drought stress response. The highest stress tolerance index (STI) was observed for TRL, NRT, NRF and NRC and compared to other traits (Table 1), implying that those traits might largely contribute to adjusting roots to the water-deficit environment.

**Table 1:** Descriptive statistics and analysis of variance (ANOVA) results for phenotypic root architectural traits of wheat under natural field-based control and drought stress environments. The plots under the rainout shelter were irrigated by a moveable overhead sprinkler which was adjusted to deliver 5 L/m^2^ of water per day. Drought stress was imposed by withholding water from tiller initiation to flowering stages, whereas control plots were under natural rainfed conditions

Traits and unit	Control						Drought					STI	H^2^	Plasticity (%)	ANOVA				Plasticity
	Min	Max	Mean	SD	CV		Min	Max	G	SD	CV				G	T	G×T		G
TRL (cm)	24.29	242.36	106.53	49.72	46.67		40.27	273.14	133.14	52.07	39.11	1.27	0.76	44.22	***	***	***		
RSA (cm^2^)	6.52	77.59	32.01	16.37	51.12		5.35	58.56	25.64	11.54	45.00	0.84	0.87	-0.86	***	***	***		***
RAD (mm)	0.51	1.38	0.94	0.18	18.95		0.27	0.96	0.61	0.13	22.28	0.65	0.70	-34.30	*	***	***		***
NRT (count)	43.00	558.00	234.86	118.95	50.65		58.00	943.00	404.11	198.49	49.12	1.69	0.74	91.52	***	***	***		***
NRF (count)	90.00	1082.00	462.48	230.36	49.81		108.00	1460.00	603.31	313.70	52.00	1.28	0.76	52.27	***	***	***		***
NRC (count)	1.00	71.00	28.32	16.27	57.43		3.00	202.00	74.63	48.51	65.00	2.62	0.73	219.73	***	***	***		***
RV (cm^3^)	0.08	1.96	0.76	0.43	56.77		0.04	1.00	0.40	0.22	53.36	0.54	0.76	-32.05	***	***	***		***

NB; the phenotypic value of all root phenotypic traits represents the mean of all accessions. *, p < 0.05; ***, p < 0.001. The abbreviations: Min, minimum; Max, maximum; SD, standard deviation; CV, coefficient of variation; NS, non-significant; TRL, total root length; RSA, root surface area; RAD, root average diameter; NRT, number of root tips; NRF, number of root forks; NRC, number of root crossings, RV, root volume; H^2^, broad-sense heritability and STI, stress tolerance index

Similarly, the calculated stress plasticity for TRL (+ 44.22), NRT (+ 91.52), NRF (+ 52.27) and NRC (+ 219.73) showed increasing trends, whereas the RSA (-0.86), RAD (-34.3) and RV (-32.05) were reduced under water-deficit stress than control conditions, respectively (Table 1). Further, ANOVA revealed significant genotypic differences (*P* > 0.05) for all of the calculated plasticity traits (Table 1). Pearson’s product-moment correlation based on genotype mean under drought treatment showed that TRL is highly and significantly correlated with NRF (0.92) and NRT (0.94), NRC with NRF (0.95) and RV with RSA (0.96), suggesting that RSA traits maintain an interconnection to accommodate the plant root system to drought stress. In contrast, RAD showed a negative correlation with the majority of the traits, except RSA and RV. However, RSA with NRF and TRL did not show any significant correlations (Fig. S1).

### Genetic association mapping and identification of candidate plasticity loci for drought tolerance

**Fig. 1:**
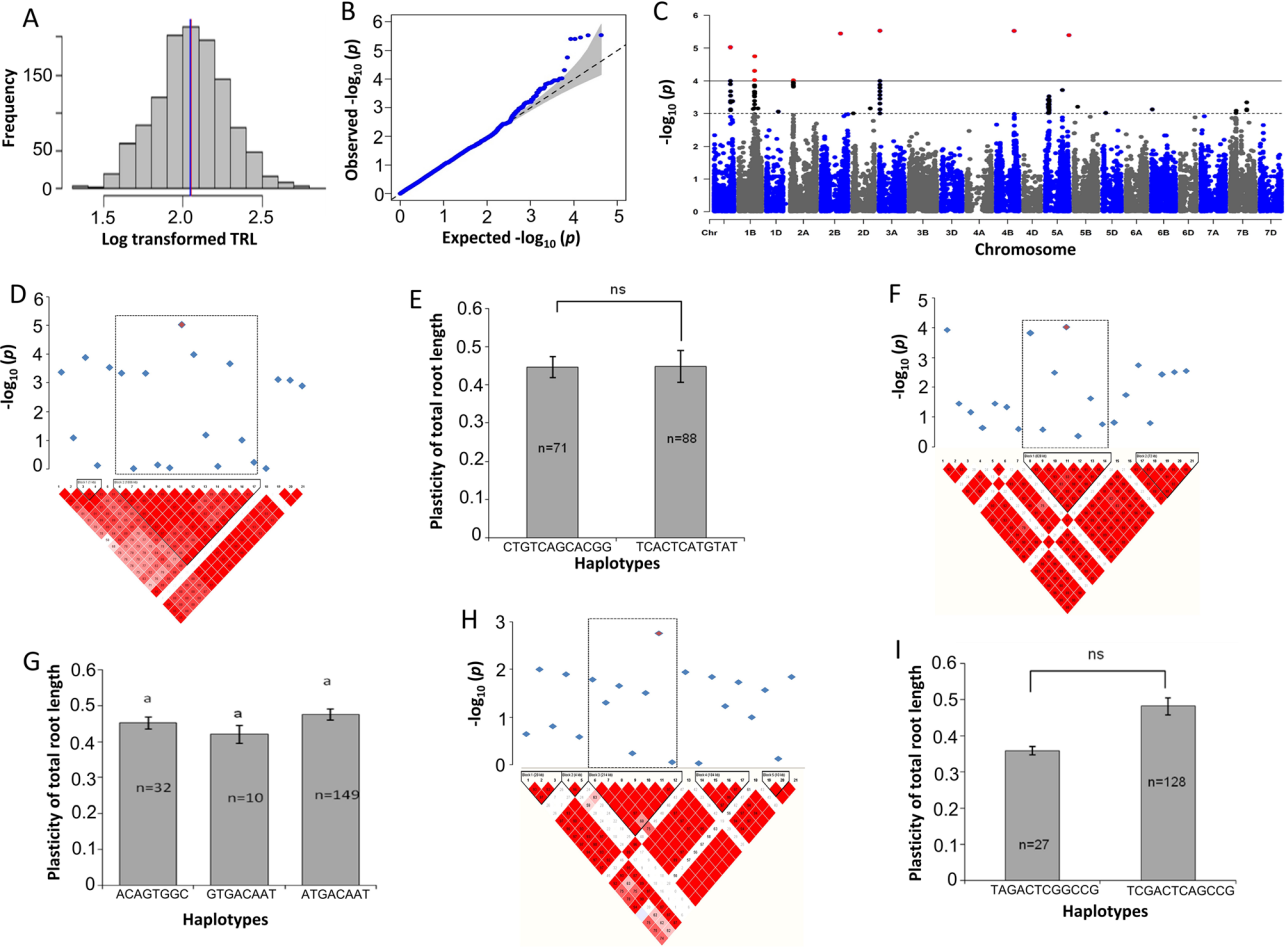
Marker-trait associations (MTAs) for the plasticity of total root length (pTRL). **a** Histogram plot highlighted the frequency distribution of log-transformed data of total root length (TRL). The blue and red color lines in the middle of plot indicate the mean and median of the data set, respectively. **b** Quantile-Quantile (Q-Q) plot indicates the efficiency of GWAS *P*-values of PRL, Y-axis: observed -log_10_ (*P*-value) and X-axis expected –log_10_ (*P*-value). **c** Rectangular Manhattan plot from association mapping of pTRL with a mixed linear model (MLM) considered the kinship and population structure matrix, Y-axis: -log_10_ (*P*-value) and X-axis: the entire 21 chromosomes of the wheat genome. The red SNPs above the black line indicated the significant SNPs which passed the threshold level at *P* ≤ 0.0001. The black SNPs above the dotted black line represented all the SNPs that did not reach the threshold level. **d, f** and **h** The linkage disequilibrium (LD) map expressing the peak region on chromosome 1 A, 2 and 3 A, respectively. Pair-wise LD map between SNP markers is denoted by *D'* values, dark red represents 1, whereas white for 0. The region surrounded by the dark dotted line represents LD blocks that harbor significant SNPs. **e, g** and **i** Phenotypic comparison of the haplotype groups established for the significant SNPs as detected by LD block. Different letters indicate statistical difference at *P* < 0.05, *n* indicates the number of genotypes representing each specific haplotype

**Fig. 2:**
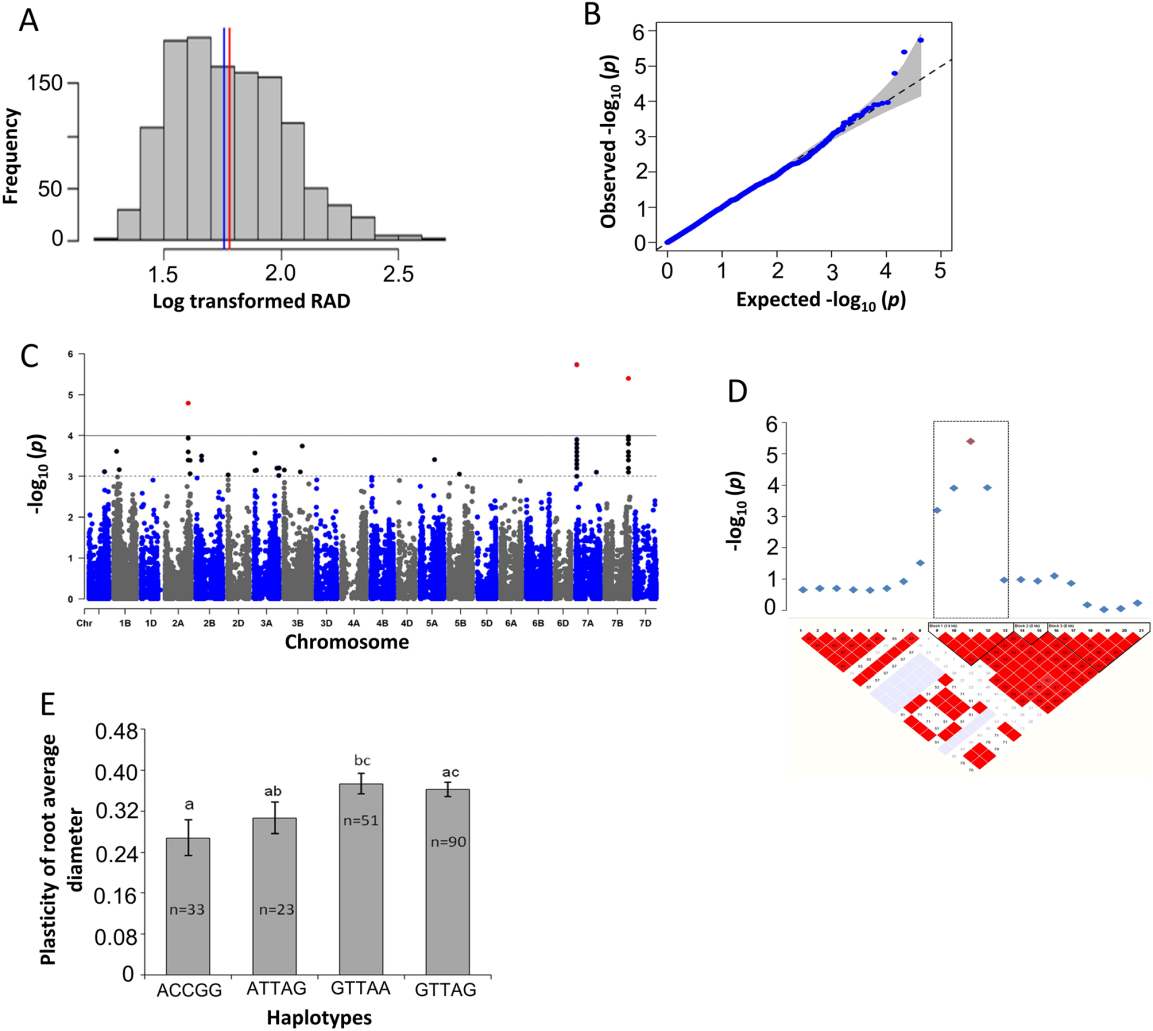
Marker-trait associations (MTAs) for the plasticity of root average diameter (pRAD). **a** Histogram plot highlighted the frequency distribution of log-transformed data of root average diameter (RAD). The blue and red color lines in the middle of the plot indicate mean and median of the data set, respectively. **b** Quantile- Quantile (Q-Q) plot indicates the efficiency of GWAS *P*-values of RAD, Y-axis: observed -log_10_ (*P*-value) and X-axis expected –log_10_ (*P*-value). **c** Rectangular Manhattan plot from association mapping of pRAD with a mixed linear model (MLM) considered the kinship and population structure matrix, Y-axis: -log_10_ (*P*-value) and X-axis: the entire 21 chromosomes of the wheat genome. The red SNPs above the black line indicated the significant SNPs which passed the threshold level at *P* ≤ 0.0001. The black SNPs above the dotted black line represented all the SNPs that did not reach the threshold level. **d** The linkage disequilibrium (LD) map expressing the peak region on chromosome 7B. Pair-wise LD map between SNP markers is denoted by *D'* values, dark red represents 1, whereas white for 0. The region surrounded by the dark dotted line represents LD block that harbor significant SNPs. **e** Phenotypic comparison of the haplotype groups established for the significant SNPs as detected by LD block. Different letters indicate statistical difference at *P* < 0.05, *n* indicates the number of genotypes represents each specific haplotype

**Fig. 3:**
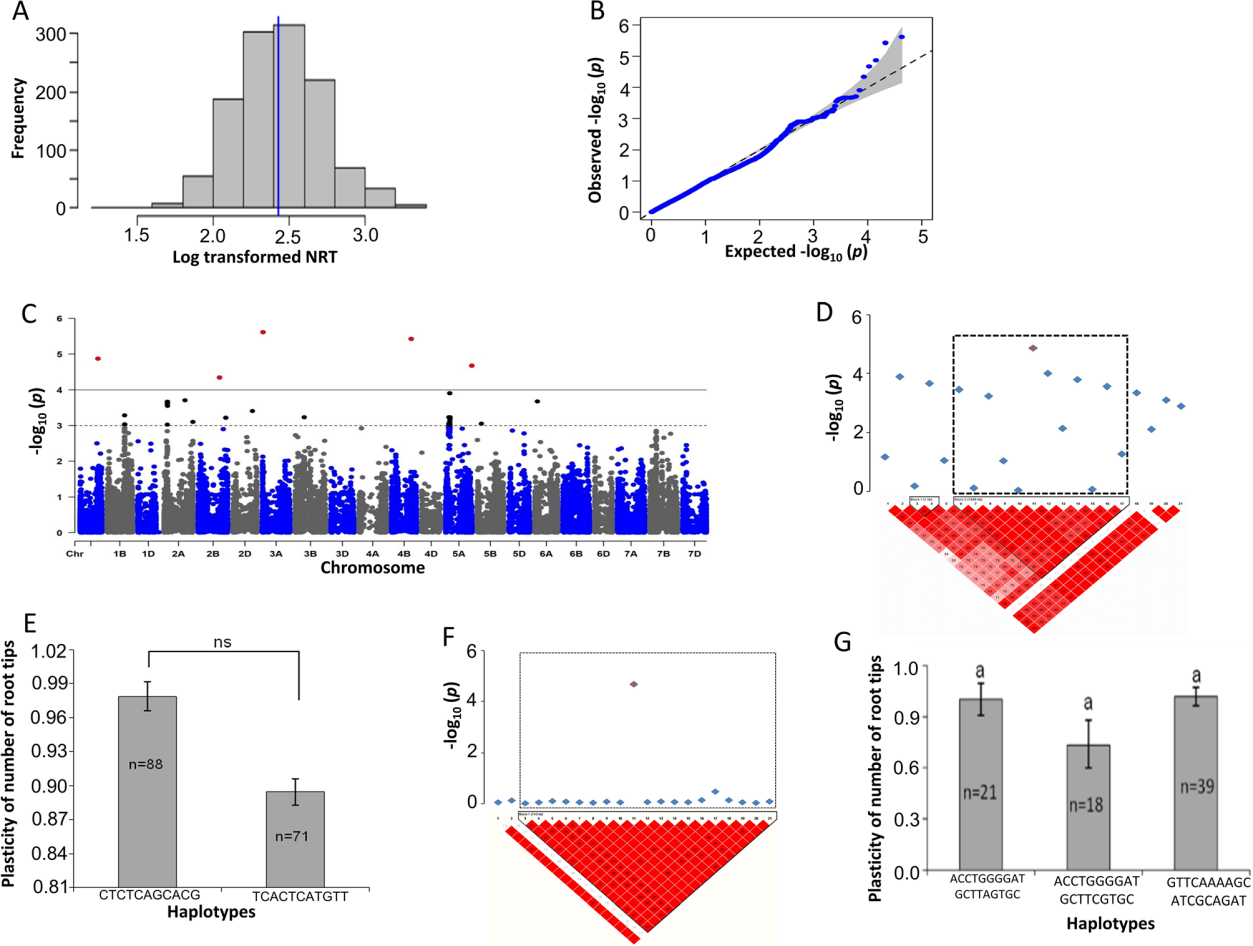
Marker-trait associations (MTAs) for the plasticity of number of root tips (pNRT). **a** Histogram plot highlighted the frequency distribution of log-transformed data of the number of root tips (NRT). The blue and red color lines in the middle of plot indicate mean and median of the data set, respectively. **b** Quantile- Quantile (Q-Q) plot indicates the efficiency of GWAS *P*-values of RAD, Y-axis: observed -log_10_ (*P*-value) and X-axis expected –log_10_ (*P*-value). **c** Rectangular Manhattan plot from association mapping of pNRT with a mixed linear model (MLM) considered the kinship and population structure matrix, Y-axis: -log_10_ (*P*-value) and X-axis: the entire 21 chromosomes of the wheat genome. The red SNPs above the black line indicated the significant SNPs which passed the threshold level at *P* ≤ 0.0001. The black SNPs above the dotted black line represented all the SNPs that did not reach the threshold level. **d**, and **f** The linkage disequilibrium (LD) map expressing the peak region on chromosome 1 and 5 A, respectively. Pair-wise LD map between SNP markers is denoted by *D'* values, dark red represents 1, whereas white for 0. The region surrounded by the dark dotted line represents LD block that harbor significant SNPs. **e** and **g** Phenotypic comparison of the haplotype groups established for the significant SNPs as detected by LD block. Different letters indicate statistical difference at *P* < 0.05, *n* indicates the number of genotypes representing each specific haplotype

To identify SNPs, the association mapping was conducted based on root morphology traits in response to drought stress and plasticity as a trait. Based on the calculated drought stress plasticity, all evaluated traits, e.g. plasticity of the total root length (pTRL), plasticity of the average root diameter (pRAD) and plasticity of the number of root tips (pNRT), demonstrated a significant association with SNPs (Figs. 1, 2 and 3). To minimize the false-positive results of the markers to trait association, an MLM was used with five PC and kinship matrices, as previously described by [31]. Based on false discovery rate (FDR)-adjusted threshold level (–log_10_*P* > 4.0), a total of 25 SNPs were identified for plasticity-related traits (pTRL, pRAD and pNRT) and RSA, NRF and RV traits in response to drought stress (Table 2). For other traits, very weak associations were observed as revealed by –log_10_*p-*values and quantile-quantile (Q–Q) plots (Figs. S2-S5). Next, LD analysis was performed based on significant SNPs to define a region containing plausible candidate genes. A total of 38 blocks harbouring 235 putative genes were defined (Table 3, Table S1). Significant markers and SNP positions were located on chromosomes 1 A, 2 and 5 A that were more likely associated with the RSA response during drought conditions, such as those responsible for water deprivation, root hair, and root cell differentiation (chromosome 1 A), in response to environmental stress (chromosome 2 A), and phenotypic switching and cellular response to heat (chromosome 5 A) (Table S1; Fig. S6). Interestingly, candidate genes were found to be related to plasticity responses, indicating their putative relationships towards drought stress (Table 4); therefore, prioritized the plasticity traits in detail.

**Table 2:** Summary of all significant single nucleotide polymorphic (SNP) markers identified by GWAS in response to drought stress and plasticity

Trait	SNP markers	Chr.	MAF	Alleles	-log_10_*p*-value	r^2^
pTRL	AX-490,522,663	1 A	0.422	C:A	5.389180199	0.135
	AX-476,020,090	1B	0.283	G:T	4.308803537	0.088
	AX-473,530,929	1B	0.185	C:T	4.023214569	0.080
	AX-89,768,547	2 A	0.19	C:T	4.015337694	0.098
	AX-585,941,635	2B	0.195	T:G	5.44267329	0.136
	AX-20,836,050	3 A	0.132	T:G	5.525885763	0.138
	AX-526,932,489	4B	0.213	A:G	5.520611864	0.138
	AX-704,835,640	5 A	0.458	A:G	5.391580949	0.134
pRAD	AX-690,934,270	2 A	0.075	C:T	4.724088353	0.077
	AX-31,875,912	7 A	0.111	T:G	5.737552483	0.084
	AX-700,388,976	7B	0.167	T:C	5.412450212	0.077
RSA	AX-814,183,606	3B	0.276	T:C	4.98699415	0.105
	AX-814,356,941	3B	0.166	G:T	4.337157206	0.089
	AX-479,202,697	5 A	0.438	T:C	4.667440359	0.119
	AX-549,850,407	5D	0.4	A:G	4.012664497	0.082
NRF	AX-65,417,557	2B	0.464	C:T	4.016279017	0.081
	AX-243,102,306	2B	0.176	A:C	4.039619611	0.099
pNRT	AX-490,522,663	1 A	0.422	C:A	4.870631037	0.233
	AX-579,774,126	2B	0.214	T:G	4.341900688	0.233
	AX-20,836,050	3 A	0.132	T:G	5.611405107	0.239
	AX-527,283,513	4B	0.198	A:G	5.424745099	0.234
	AX-705,374,739	5 A	0.458	A:G	4.674389488	0.233
RV	AX-695,555,707	3B	0.37	A:G	4.522651694	0.083
	AX-814,183,606	3B	0.276	T:C	4.529192156	0.086

The SNPs with –log_10_ (*P*-value) ≥ 4.0 (threshold set by 5% FDR correction) are listed together with the corresponding trait. Abbreviation: Chr., chromosome; MAF, minor allele frequency; r^2^, marker r^2^ values; A, adenine; G, guanine; T, thymine; C, cytosine; TRL, total root length, RAD, root average diameter; RSA, root surface area; NRF, number of root forks; NRT, number of root tips and RV, root volume

**Table 3:** List of the number of single nucleotide polymorphisms (SNPs) and candidate genes identified by GWAS based on significant marker-trait associations (MTAs) and linkage disequilibrium (LD) block recognized from the putative regions in response to drought stress and plasticity

Trait	Chr.	LD block	Number of SNPs	Putative Region (bp)		Number of genes
				Start	End	
pTRL	1 A	Block 2	12	4.89E + 08	491,149,320	19
	1B	Block 1	31	4.72E + 08	478,722,611	35
	2 A	Block 1	7	89,597,473	90,425,823	5
	2B	Block 3	8	5.82E + 08	587,815,112	8
	3 A	Block 3	7	20,741,217	21,069,728	10
	4B	Block 1	18	5.27E + 08	529,334,216	3
	5 A	Block 1	19	7.05E + 08	705,565,704	3
pRAD	2 A	Block 2	9	6.91E + 08	691,181,098	8
	7 A	no block	1	30,875,862	32,875,862	6
	3B	Block 3	11	8.14E + 08	815,504,335	12
RSA	5 A	Block 2	9	4.79E + 08	479,203,284	4
	5D	Block 3	3	5.5E + 08	549,852,162	1
	2B	Block 1	10	65,225,853	65,808,631	4
NRF	2B	Block 1	21	2.38E + 08	244,534,860	24
	1 A	Block 2	12	4.89E + 08	491,149,320	20
pNRT	2B	Block 2	4	5.79E + 08	580,020,356	9
	3 A	Block 3	7	20,741,217	20,955,640	9
	4B	Block 2	17	5.27E + 08	529,334,216	14
	5 A	Block 1	19	7.05E + 08	705,565,704	9
	3B	Block 1	15	6.94E + 08	696,288,703	14
RV	3B	Block 3	11	8.14E + 08	815,504,335	18

The significant single nucleotide polymorphisms (SNPs) which does not belong to an LD block, a 1Mbp window on either side of significant SNP was considered to search putative candidate genes. The same chromosome with the same colour shade represents their common sharing of the same putative region for different traits. Abbreviation: pTRL, plasticity of total root length; pRAD; plasticity of root average diameter; RSA, root surface area; NRF, number of root forks; pNRT, plasticity of number of root tips; RV, root volume; Chr, chromosome and bp, base pair

**Table 4:** Short-list of plasticity-responsive candidate genes based on their functional involvement in drought tolerance mechanisms

Traits	Chr.	Gene ID	Gene size	Gene annotation	
				Molecular function	Biological function
pTRL	1 A	TraesCS1A02G295000	1,549	protein self-association-unfolded protein binding (GO:0006950), abscisic acid binding-signaling receptor activity (GO:0009725),	response to heat- response to reactive oxygen species-response to salt stress (GO:0006950), abscisic acid-activated signaling pathway (GO:0009725)
		TraesCS1A02G295400	629	water channel activity (GO:0009414), hydrolase activity(GO:0005886)	response to water deprivation (GO:0009414), carbohydrate metabolic process(GO:0005886)
		TraesCS1A02G296200	4,331	structural molecule activity (GO:0006888)	response to freezing (GO:0050826), intracellular protein transport-vesicle-mediated transport (GO:0006888)
		TraesCS1A02G296300	5,040	potassium ion leak channel activity(GO:0016021), actin filament binding-ATP binding(GO:0048765)	root hair elongation-vesicle transport along actin filament- root hair cell differentiation (GO:0048765) , lateral root development (GO:0048527)
	1B	TraesCS1B02G269100	1,321	protein self-association-unfolded protein binding (GO:0006979), growth factor activity-growth hormone receptor binding-hormone activity(GO:0060416)	response to heat -response to reactive oxygen species -response to salt stress (GO:0006979), lateral root development (GO:0048527), positive regulation of growth-response to growth hormone **(**GO:0060416)
		TraesCS1B02G272100	3,720	water channel activity- (GO:0006833), cellular response to water deprivation (GO:0042631), hydrolase activity (GO:0048046)	Transport-water transport (GO:0006833), apoplast (GO:0048046)
		TraesCS1B02G272900	4,291	auxin-activated signaling pathway-transmembrane transport (GO:0009926)	Auxin signaling pathway, auxin polar transport (GO:0009926)
		TraesCS1B02G269400	3,006	protein self-association-unfolded protein binding(GO:0009651), hydrolase activity, hydrolyzing O-glycosyl compounds (GO:0005886)	response to heat-response to reactive oxygen species (GO:0009651), response to cold (GO:0009409)
		TraesCS1B02G272000	2,745	water channel activity(GO:0009414)	response to water deprivation(GO:0009414), response to cold (GO:0009409)
	2 A	TraesCS2A02G144900	1,511	heme binding-metal ion binding-peroxidase activity(GO:0009505), chlorophyll binding (GO:0009628)	response to environmental stresses(GO:0009505), response to abiotic stimulus (GO:0009628)
		TraesCS2A02G145000	1,181	chlorophyll binding (GO:0009628)	light harvesting in photosystem I-response to light stimulus (GO:0009628) peroxidase activity-environmental stress (GO:0004601)
	2B	TraesCS2B02G409800	1,306	actin-dependent ATPase activity-actin filament binding-ATP binding- (GO:0048765), voltage-gated ion channel activity (GO:0009913)	actin filament organization-root hair elongation-vesicle transport along actin filament-root hair cell differentiation (GO:0048765)
		TraesCS2B02G410400	4,291	protein self-association-unfolded protein binding (GO:0006950)	response to heat- response to reactive oxygen species-response to salt stress - response to stress (GO:0006950)
	4B	TraesCS4B02G259600	3,639	ion channel binding(GO:1,903,959), protein-macromolecule adaptor activity(GO:0040008)	regulation of anion transmembrane transport (GO:1,903,959),response to starvation-positive regulation of cell growth (GO:0040008)
	5 A	TraesCS5A02G551700	939	ATPase activity- ATP binding -unfolded protein binding(GO:0034605), DNA-binding transcription factor activity-sequence-specific DNA binding(GO:0009751)	cellular response to heat (GO:0034605), hyperosmotic salinity, and hormone response(GO:0009751)
pRAD	7 A	TraesCS7A02G064700	3,204	hydrolase activity-methyl jasmonate esterase activity-methyl salicylate esterase activity(GO:0009694), transcription regulatory region DNA binding	cellular response to auxin stimulus (GO:0071365), jasmonic acid-mediated signaling pathway (GO:0009864)
	7B	TraesCS7B02G432200	3,706	potassium ion leak channel activity(GO:0016021)	potassium ion transmembrane transport-stabilization of membrane potential(GO:0016021) , response to light intensity (GO:0009642)
pNRT	1 A	TraesCS1A02G295400	629	water channel activity(GO:0009414), chlorophyll-binding (GO:0009579), hydrolase activity, hydrolyzing O-glycosyl compounds(GO:0005886), potassium ion leak channel activity (GO:0005774)	response to water deprivation (GO:0009414), photosynthesis, light harvesting in photosystem I- response to the light stimulus (GO:0009579), stabilization of membrane potential (GO:0005774)
		TraesCS1A02G295500	261	protein self-association-unfolded protein binding(GO:0006950)	response to heat-response to hydrogen peroxide-response to reactive oxygen species-response to salt stress-response to stress (GO:0006950)
		TraesCS1A02G295700	522	water channel activity(GO:0009414), potassium ion leak channel activity (GO:0065007), hydrolase activity, (GO:0005886)	response to water deprivation(GO:0009414), ), potassium ion transmembrane transport-stabilization of membrane potential(GO:0065007),
		TraesCS1A02G296300	5,040	actin-dependent ATPase activity- (GO:0048765, ATP binding-protein serine/threonine kinase activity-transforming growth factor-beta receptor activity, type I(GO:0004675)	root hair cell differentiation and root hair elongation (GO:0048765) , lateral root development (GO:0048527), cellular response to growth factor stimulus (GO:0004675)
	3 A	TraesCS3A02G039000	4,478	ATP binding-protein serine/threonine kinase activity (GO:0005819)	response to auxin-response to ethylene-response to gibberellin(GO:0009733), photosynthesis, light harvesting in photosystem I-response to the light stimulus (GO:0009941)
	4B	TraesCS4B02G259600	3,639	ion channel binding (GO:1,903,959), sodium-independent organic anion transmembrane transporter activity (GO:0098656), protein-macromolecule adaptor activity(GO:0040008), potassium ion leak channel activity(GO:0005774), voltage-gated chloride channel activity(GO:0008308)	cellular response to starvation-positive regulation of cell growth-positive regulation of protein serine/threonine kinase activity-regulation of cell size-regulation of growth (GO:0040008) , potassium ion transmembrane transport-stabilization of membrane potential-vacuolar membrane (GO:0005774), voltage-gated chloride channel activity(GO:0008308)
		TraesCS4B02G260200	2,261	potassium ion leak channel activity(GO:0016021), abscisic acid binding-protein phosphatase inhibitor activity-signaling receptor activity (GO:0050896)	potassium ion transmembrane transport-stabilization of membrane potential (GO:0016021), response to stimulus (GO:0050896)
	5 A	TraesCS5A02G551700	939	hydrolase activity(GO:0005886), ATPase activity, (GO:0034605)	phenotypic switching (GO:0036166), cellular response to heat (GO:0034605) , hyperosmotic salinity response, response to auxin (GO:0009733)

The abbreviation: pTRL, plasticity of total root length; pRAD; plasticity of root average diameter; pNRT, plasticity of number of root tips; Chr, chromosome and GO, gene ontology

#### Plasticity of the total root length

The association mapping of the pTRL was performed from TRL mean values, yielding significant marker-trait associations (MTAs) (Fig. 1). The log-transformed TRL data demonstrated the normal distribution with equal mean and median values (Fig. 1a). The Q-Q plot showed that the observed *P*-value of pTRL deviated from the expected *P*-value (Fig. 1b). Manhattan plot revealed that nine SNPs passed the threshold level at 5% FDR. Those MTAs occurred on chromosome 1B, one consistent peak consisting of three significant SNPs and on other chromosomes 1A, 2 A, 3 A, 4B and 5 A, each peak consisting of only one SNP (Fig. 1c). The LD block heat-map indicated that significant SNP markers of chromosome 1A (AX-490,522,663) grouped with other 11 SNP markers on a major LD block (Fig. 1d, e). The significant SNP of chromosome 2 A (AX-89,768,547) was grouped with other six SNPs on a major LD block (Fig. 1f). The major LD block of chromosome 3 A with significant SNP was grouped with six other SNPs (Fig. 1g).

The LD block on chromosome 1 A contained two main haplotypes, CTGTCAGCACGG and TCACTCATGTAT, which belonged to 71 and 88 cultivars, respectively. For both haplotypes, no significant differences were observed for pTRL based on Student’s *t*-test (Fig. 1d, e). The LD block of chromosome 2 A contained three main haplotypes, 32 cultivars possessing ACAGTGGC, 10 containing GTGACAAT and 149 harbouring ATGACAAT and all three haplotypes did not differ from each other in their association values with pTRL (Fig. 1f, g). Another LD block on chromosome 3 A also formed two main haplotypes, TAGACTCGGCCG within 27 genotypes and TCGACTCAGCCG 128 cultivars, and also showed a non-significant difference for the pTRL trait (Fig. 1 h, i).

The major LD blocks for the pTRL contained putative candidate genes with annotation in multiple biological processes, such as response to the heat and reactive oxygen species, water deprivation, responses to environmental stress and abiotic stimulus, cellular response to auxin signaling pathway, root hair elongation and lateral root development (Table 4).

### Plasticity of the average root diameter

The association mapping conducted on pRAD has shown significant MTAs (Fig. 2). The log-transformed data revealed a normal distribution with equal mean and median values (Fig. 2a). The Q-Q plot indicated the observed *P*-value of pRAD that deviated from the expected *P*-value (Fig. 2b). Manhattan plot suggested that three SNPs satisfied the threshold level at 5% FDR, which all relied on chromosomes 2A, 7 A and 7B (Fig. 2c). The LD block heat-map indicated significant SNP markers of chromosome 7B (AX-700,388,976) lineage with their four neighbour SNP markers on a major LD block (Fig. 2d). The LD block on chromosome 7B established four main haplotypes, in which ACCGG belonged to 33 cultivars, ATTAG carried 23 cultivars, GTTAA belonged to 51 cultivars and GTTAG possessed 90 cultivars for pRAD traits (Fig. 2e). The Student’s *t*-test analysis for these four haplotypes showed that ACCGG and GTTAA haplotypes had significant differences for the trait, and when compared to the haplotypes, both showed non-significant differences for pRAD (Fig. 2e).

The LD block for pRAD with significant SNP markers harboured candidate genes associated with biological processes, such as cellular response to auxin stimulus, jasmonic acid-mediated signaling pathway, potassium ion transmembrane transport, stabilized membrane potential and response to the light intensity with the molecular activity of hydrolysis (Table 4).

### Plasticity of the number of root tips

To perform the association mapping, stress plasticity was calculated using the NRT values, which revealed significant MTAs (Fig. 3). The normality distribution of NRT displayed by the log-transformed data contained exactly equal mean and median values (Fig. 3a). The Q-Q plot revealed the observed *P*-value of pNRT that deviated from the expected *P*-value (Fig. 3b). The Manhattan plot implied that five SNPs confirmed the threshold level at 5% FDR. These five SNPs were found on chromosomes 1 A, 2 A, 3A, 4B and 5 A (Fig. 3c). Significant SNP markers on chromosome 1 A (AX-490,522,663) established an LD block by linkage with the other 11 SNP markers (Fig. 3d). The LD block of significant SNP markers on chromosome 5 A (AX-705,374,739) formed a linkage with the other 18 neighbour SNPs (Fig. 3e). The LD block on chromosome 1 A contained two main haplotypes, 88 cultivars carrying CTGTCAGCACG, whereas 71 cultivars possessed TCACTCATGTT and the Student’s *t*-test analysis showed that both haplotypes had non-significant differences for the pNRT (Fig. 3d). The LD block on chromosome 5 A contained three main haplotypes, with ACCTGGGGATGCTTAGTGC belongs to 21 cultivars, ACCTGGGGATGCTTCGTGC on 18 cultivars, and GTTCAAAAGCATCGCAGAT on 39 cultivars, which all exhibited non-significant differences for pNRT (Fig. 3d).

The LD block for pNRT containing candidate genes showed functional associations in different biological functions, such as responses to water deprivation, response to heat, response to reactive oxygen species, root hair cell differentiation, root hair elongation, lateral root development, response to auxin and other hormones, phenotypic switching, hyperosmotic salinity response and also molecular activity, such as water channel, hydrolysis and ATPase (Table 4).

#### Expression analysis of identified candidate genes under drought stress

**Fig. 4:**
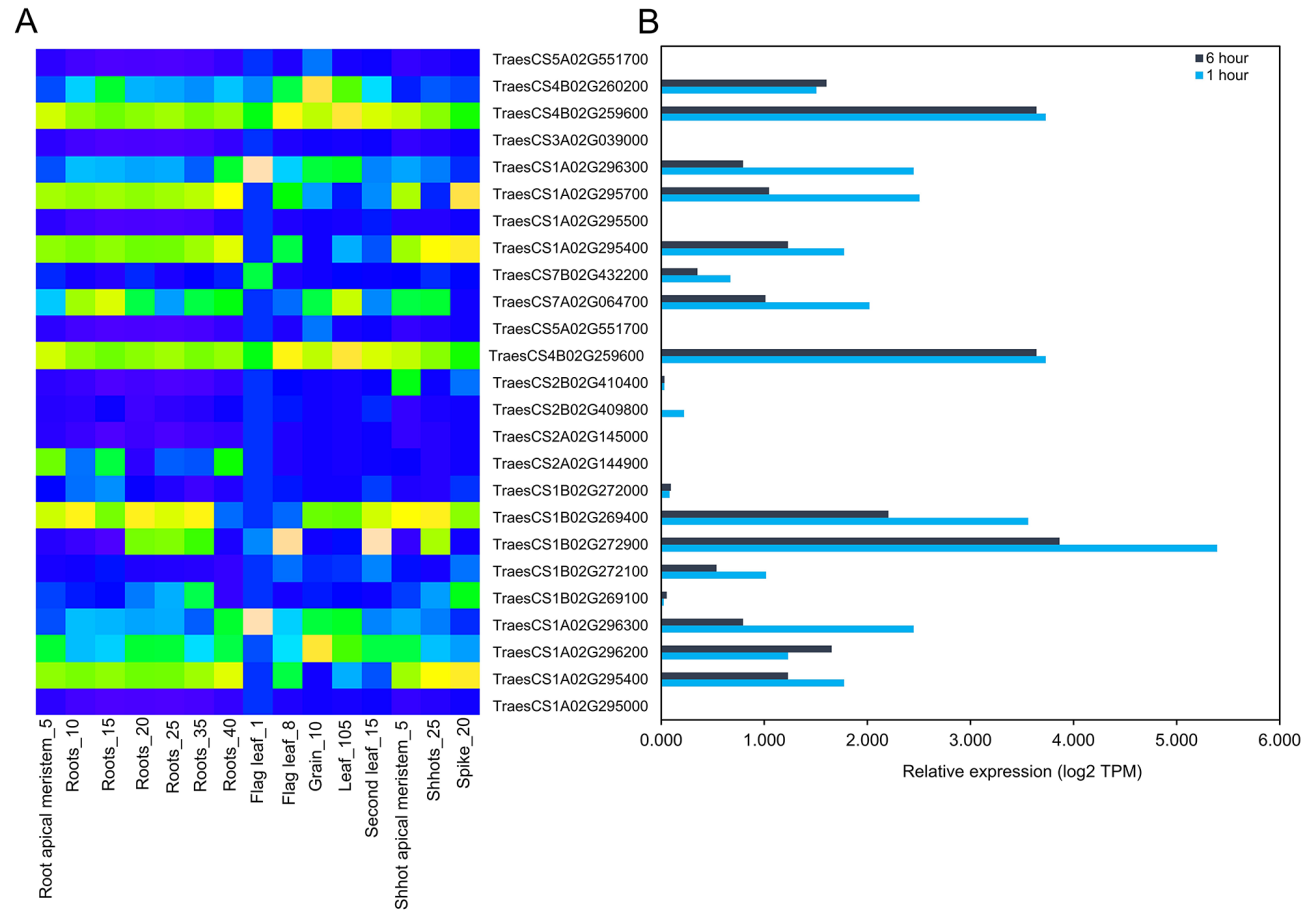
Transcript expression patterns of selected plasticity-responsive candidate genes for root growth and drought tolerance. **a** Expression within different tissues of wheat represented as root apical meristem_5 (tillering stage), roots_10 (flag leaf stage), roots_15 (30% spike), roots_20 (14-days old), roots_25 (seven leaf stage), roots_30, roots_35 and roots_40 (fifth leaf stage), flag leaf_1 (milk grain stage), flag leaf_8 (12 dpa), grain_10 (ripening stage), leaf_105 (9-days old), second leaf_15 (17-days old), shoot apical meristem_5 (tillering stage), shoots_25 (2-days old) and spike_20 (flag leaf stage). Deep blue color indicates lower and deep coral color indicates higher expression values (log2 TPM). **b** Expression patterns of candidate genes under 1 h (deep sky blue color bar) and 6 h (black color bar) of drought treatments. RNA-seq data were curated from the Wheat Gmap database and are represented by a heatmap of transcripts per kilobase million (TPM) values. The expression data is also provided in Supplementary Table S2 and S3

Expression levels of identified candidate genes were estimated using the WheatGmap browser (https://www.wheatgmap.org) [32]. We observed a wide range of transcript expression for these genes in different developmental stages, including multiple root growth stages (Fig. 4a). Among the top 25 short-listed plasticity-associated candidate genes based on their functional annotations in drought (Table 4), the majority of candidate genes were found to be highly expressed in roots at different developmental stages (Fig. 4a, Table S2), indicating that they might be involved in root growth and development. Next, their expression levels were determined using the above-mentioned WheatGmap database after 1 and 6 h of drought imposition (Fig. 4b). Varying expression levels were observed among selected candidate genes, whereas eight genes were stably and largely expressed in response to both 1 and 6 h of drought treatments (Fig. 4b, Table S3). Interestingly, these eight genes were particularly expressed in the roots and simultaneously under drought stress, predicting that these genes play vital roles in root developmental plasticity to better withstand plants’ to drought stress.

## Discussion

In wheat, root architectural traits are crucial in water and nutrient acquisition, and root growth patterns have been reported to be highly affected by soil water availability [33]. Importantly, root architectural traits are plastic in response to drought crucial for water and nutrient acquisition, anchorage and storage [18, 34]. Therefore, elaborating our knowledge on the genetic control of root architectural trait plasticity and uncovering their associated candidate genes will be useful to improve wheat productivity in water-limited areas.

To analyze the genetic components of root architectural plasticity to water availability, root system traits were phenotyped under normal watering and drought conditions by withdrawing water supply from tillering to the flowering stage of the wheat growth to identify natural genetic variations and putative genes associated with RSA plasticity. As a result of drought imposition, wheat cultivars showed significant phenotypic variations of root traits, including TRL, RAD, NRF, NRC and NRF, indicating that these traits are more responsive during water-deficit stress (Tables 1 and 27). The results obtained in this study were similar to those of a previous study on water-deficit stress conditions, showing that plants increase the root length to enter into the deep soil layers to better explore the soil and is accompanied by drought tolerance [35, 36]. The results presented here also indicated that the RAD decreased during a water shortage, whereas TRL was increased (Table 1). This might be the candidate for improving plant adaptation to drought. Reduced root diameter with greater root length has been established as a trait involving the enhancement of plant productivity during drought [37]. A narrow root diameter is beneficial for plants that can efficiently increase hydraulic conductance to minimize the root apoplastic barrier for entering water into the xylem [38–40]. Conversely, we found that the RSA and RV of the root were found to be decreased under drought conditions at the complete flowering stage (Table 1). Similarly, recent studies also reported that wheat genotypes under drought showed differential responses of root traits due to their growth stages, such as the increased RSA during the anthesis stage but reduced at the maturity stage [41], which may indicate that the RSA and RV of the root responses vary with plant growth stages. However, the NRF and NRC are also crucial root architecture traits that plays a significant role in enhancing the penetration ability of pants into the soil layers, resulting in a positive effect on accessing water and nutrients [42]. We observed that wheat genotypes increased the NRT under drought conditions than in the control condition (Table 1). Increasing the lateral root number helps plants to improve the water transport to sustain the drought stress condition and for rapid access to soil moisture [43, 44]. Pearson’s product-moment correlation heat-map showed that TRL was negatively correlated with RAD and positively correlated with NRT, NRF and NRC (Fig. S1), indicating that drought stress resulted in a decreased root diameter with an increased root length and root number that might be critical for increasing total water uptake area [45].

Before performing a GWAS, fulfilling the requirements of individuals with high genetic diversity is essential for obtaining more allelic variations [46]. The LD patterns of the 200 genotypes decayed after 19.0, 38.0 and 17.5 Mbp for A, B and D genome, respectively, indicating that the LD decay patterns of the B genome was slower than the A and B genome [47]. In this GWAS, an MLM, including the PC and kinship matrix, enabled to avoidance of false MTAs [48]. Following these approaches, a total of 25 significant SNPs harbouring 235 putative candidate genes were detected for the trait associated with plasticity, such as pTRL, pRAD and pNRT and drought-treated RSA, NRF and RV after a successful FDR correction (Tables 2 and 3), although NRC did not yield any significant SNPs. These results indicate that those root traits were controlled by a diverse set of genes to adjust to drought conditions [50]. A total of 235 possible candidate loci were identified across wheat chromosomes, which were associated with root trait responses during drought stress. (Tables 2 and 3). The majority of the SNP related to plasticity was located on chromosomes 1 A, 2 and 5 A associated with a better response for root plasticity traits under water-deficit stress (Figs. 1, 2 and 3). The abundant genomic regions in wheat for drought and root-related traits are detected on chromosomes 1 A, 2 and 5 A encompassing plausible genes upregulated during abiotic stresses [49].

The LD analysis detects neighboring and associated SNPs based on the relationship of SNPs on the adjacent stretch of genomic regions within the population; thereby, LD explains genetic variations over the population [50]. The putative candidate genes were identified based on LD blocks harbouring significant SNP markers (Tables 3 and 4). The LD-based GWAS successfully delivers chromosomal regions underlying candidate genes affecting the plant adaptation to environmental stresses [31, 51]. Next, haplotype analysis was performed for plasticity-related traits, pTRL, pRAD and pNRT. For the pTRL on chromosome 1 A, two haplotype blocks, and 2 A, and chromosome 3 A in two haplotypes were abundantly observed under drought stress conditions (Fig. 1d-i). The pRAD contained four main haplotypes on chromosome 7B, which were associated with the plasticity of RAD under drought conditions (Fig. 2d, e). Haplotype blocks of the pNRT trait on chromosome 1 A formed two and on chromosome 5 A three distinct haplotypes, respectively. Those were widely dispersed on cultivars that may be related to the pNRT under drought conditions (Fig. 3d-g). Interestingly, all adaptive loci carrying major haplotypes were found to have larger contributions to the root phenotypic plasticity under drought when compared with minor haplotypes (Figs. 1, 2 and 3), suggesting that exchanging these haplotype alleles could induce root phenotypic adaptation to drought conditions.

Candidate genes associated with pTRL under drought conditions showed biological functions on responses to heat and reactive oxygen species, water deprivation, environmental stress response and abiotic stimulus, cellular response to auxin signaling pathway, root hair elongation and lateral root development (Table 4). Another study showed that some genes can regulate the auxin signaling pathway under drought stress and assist in lateral root formation or root elongation to access more water from its surrounding environments [52]. The putative candidate genes for pRAD were associated with the cellular response to auxin stimulus, jasmonic acid-mediated signaling pathway and stabilization of membrane potential (Table 4). The genes were upregulated in response to drought for increasing cell division, tropisms, vascular differentiation and root meristem maintenance. Moreover, a jasmonic-acid-responsive gene shows an interactive function for the plant’s tolerance to abiotic stress conditions [53].

The pNRT under water-deficit conditions was putatively controlled by multiple candidate genes (Table 4). Upregulations of genes responsible for root water deprivation and abscisic-acid biosynthesis associated with auxin transport to the root tips are major factors of drought stress tolerance [54]. Overall, we short-listed 25 plasticity candidate genes showing highly putative relationships with drought (Table 4). *In silico* transcript expression analysis showed distinct expression levels of eight candidate genes in root under drought treatments in multiple root growth stages (Fig. 4). This result confirms that these eight candidate genes are particularly associated with drought stress adaptation underlying the root growth plasticity.

## Conclusion

In this study, root phenotypic traits were quantified in the global collection of winter wheat cultivars with and without water supply in the field environment. Substantial genetic variations were revealed for all of the root traits in response to drought and plasticity that determines the phenotypic responses to water availability. Further, the identified MTAs and candidate genes, especially for root phenotypic plasticity, will be useful for further functional studies in improving the wheat root systems to better withstand plants in water-deficit soils. These results also provide additional insights into the drought-induced natural root system variations within diverse wheat germplasms. These results also reveal that the elite candidate loci/genes in wheat are more responsive to root trait plasticity. Therefore, plasticity-associated markers and candidate genes could be useful for breeding tolerant wheat varieties. However, further in-depth investigation is crucial to better understand the genetic relationships between the phenotypic plasticity of root architectural traits and candidate SNPs underlying drought adaptation, which may hasten root-targeted breeding programs to develop drought-resilient wheat cultivars.

## Materials and methods

### Plant materials

In this study, a total of 200 diverse wheat cultivars were used to assess the genetic diversity of root architecture traits as described by [31] and [55]. The full names and the source of wheat cultivars are available in Siddiqui et al. [31]. Among these 200 cultivars, 60% were obtained from Germany and the others from different countries.

### Field experiment

The field experiment was conducted in the 2019–2020 wheat growing season under natural rain-fed (control) and rain-out shelter (drought stress) conditions, as previously described by [31]. The experiment was designed as a split-plot over randomized complete block design (RCBD), where treatments (control and drought) were defined as main plots. The 200 wheat cultivars were sown in a RCBD within the main plots with three replications; each plot contained one cultivar in a single row length of 0.5 m and a between-row distance of 0.21 m. Each plot contained four rows flanked by two border rows to avoid edge effects. Each plot was irrigated by movable sprinklers that deliver ~ 5.00 L/m^2^ water per day. Then, drought was induced by withholding water and closing the roof cover. Drought treatment was applied from the tillering initiation phase at the end of April 2020 (BBCH21) to the complete flowering phase around the middle of June 2020 (BBCH65). The soil water content of well-watered treatment was ranged from 0.18 to 0.20 m^3^m^3^, whereas drought-treated plots ranges from 0.063 to 0.079 m^3^m^3^ throughout the drought periods [31]. The soil texture of the experimental plot was a Haplic Luvisol derived from loamy silt [56]. Details of fertilizer application, management practices and soil moisture levels (0–30 cm) under control and drought treatment were described by [31].

### Root phenotyping using the ‘Shovelomics’ approach

The wheat root system was phenotyped by the “Shovelomics” protocol, i.e. digging out the upper part of the wheat root and determining the root architectural traits [23, 57, 58]. The root systems from three individual plants per plot were excavated at BBCH65 using a shovel to maintain a distinct depth of 27 cm (distance from the cutting edge to the shoulder of a shovel) at a distance of ~ 0.2 m away from the plant base to avoid root destruction from both the control and drought-stressed plants [21]. The lumps of excavated soil containing the roots were dissolved by submerging them in a freshwater bucket until the soil was removed from the roots (Fig. S7). Thereafter, the roots were gently washed to remove the remaining soil particles and rinsed with clean water. After fine washing, the roots were separated from the shoot by cutting at the root-shoot junction. The clean and fresh roots were preserved with 50% alcohol in a plastic pot. Then, the preserved roots were scanned using an Epson scanner (Perfection LA24000) with a resolution of 600 dots per inch and root images were analyzed using the WinRhizo software (Regent Instruments Inc., Quebec, Canada) to record the root architectural traits [17, 57].

### Statistical analysis

The collected phenotypic data on root system traits were analyzed using the R software version 3.6.1 [59]. Before analysis, extreme outliers we removed based on the following criteria of the mean of all accessions ± 3 standard deviation (SD), as described by [60]. Then, data normality was tested following the histogram evaluation, Shapiro-Wilk test and box plot in the R studio. For the descriptive study, a two-way ANOVA for root system traits such as; total root length (TRL), root average diameter (RAD), root surface area (RSA), number of root tips (NRT), number of root crossings (NRC), number of root forks (NRF) and root volume (RV) was conducted. During the ANOVA analysis, cultivar and treatment effects were considered as fixed effects with the interaction, whereas block was considered as a random effect [33]. Descriptive statistics such as the mean, median, mode, min, max, coefficient of variation, and SD, were analyzed. Besides, Pearson’s pairwise correlations (r) were calculated for all RSA traits to determine the correlation between phenotypes using statgraphics version 18.1.13 software. Heritability was estimated using broad-sense heritability (H^2^) and calculated using the following formula [61]: H^2^ = V_G_/(V_G_+V_E_/r), where V_G_ is the estimation of genetic variance, V_E_ is the estimation of error variance for each treatment and r is the number of replications of each cultivar. Stress plasticity of the root system architectural traits was calculated using the following equation: (P)= (WW-WS)/WW, where WS is water stress and WW is well-watered as described by [18]. Moreover, STI of root traits of all cultivars was estimated by taking the phenotypic values under water stress and well-watered conditions using the following equation: STI= (WS × WW)/(mean WW)^2^ where WS is water stress and WW is well-watered, following [62].

### Genome-wide association study

The GWAS was conducted for seven root architectural traits (TRL, RAD, RSA, NRT, NRC, NRF, and RV) under water-stressed conditions. A total of 24,216 SNP markers were obtained employing the genomic DNA extraction process [63] which was used to evaluate the genetic variation of the root traits. The association mapping was performed using the TASSEL software 5.2.54 [64], following the mixed linear model (MLM) including five principal components (PC) and a kinship matrix [33]. To set the significant threshold, a 5% FDR was calculated using the ‘*q*value’ package of the R software [65]. The Q-Q plot and rectangular Manhattan plots were prepared using the CM plot package of R [66]. Significant SNP markers were defined based on the FDR threshold and satisfy the requirements of FDR *q-*value < 5% considered as true positive [33].

### Candidate gene selection and expression analysis

For each root trait, the Plink formats of the data and LD were analyzed using Haploview 4.2 to identify the candidate loci [67]. The defined LD block heat map was determined based on the D´ value in the upper confidence bounds exceeded 0.98 and lower bounds of > 0.7 [68]. To compare the haplotype belonging to a block with significant SNP markers, the student’s t-test (two samples assuming equal variances) and Tukey’s test > 2 haplotype comparisons were performed. LD blocks with highly significant SNP markers were expected to carry putative candidate loci. Significant SNPs that did not establish haplotype blocks, i.e. the genes close to these loci (1 Mbp from both sides) were considered putative candidate genes as stated by [53]. The gene annotation and ontology were conducted in the wheat URGI database [69]. To locate significant SNPs on various chromosomes, a map chart was generated following [70]. Further, expression profiles of the drought-associated candidate genes were curated using the publicly available RNA-seq data [32].

## Electronic supplementary material

Below is the link to the electronic supplementary material.

Supplementary Material 1

## Data Availability

All data generated or analyzed during this study are included in this published article and its supplementary information files. The accession and genetic polymorphisms data are available here: 10.1038/s41598-021-85226-1.

## Abbreviations

GWAS: Genome wide association studies
LD: Linkage disequilibrium
QTL: quantitative trait loci
SNPs: single nucleotide polymorphisms
pTRL: plasticity of the total root length
pRAD: plasticity of the average root diameter
pNRT: plasticity of the number of root tips
MTAs: marker-trait associations
RCBD: randomized complete block design
TRL: total root length
RAD: root average diameter
RSA: root surface area
NRT: number of root tips
NRC: number of root crossings
NRF: number of root forks
RV: root volume
STI: stress tolerance index
MLM: mixed linear model
