# Supplementary material for: Genetic dissection of root architectural plasticity and identification of candidate loci in response to drought stress in bread wheat
Source: BMC Genom Data. 2023 Jul 26;24:38. doi: 10.1186/s12863-023-01140-7 (PMC10373353; doi:10.1186/s12863-023-01140-7)
Supplement: Supplementary file 1 — Supplementary Material 1 [file 12863_2023_1140_MOESM1_ESM.pdf]

Supplementary information:

Title: Genetic dissection of root architectural plasticity and identification of candidate loci in response to drought stress in bread wheat

Authors: Md. Nurealam Siddiqui, Melesech T. Gabi, Mohammad Kamruzzaman, Abebaw M. Ambaw, Tesfaye J. Teferi, Said Dadshani, Jens Léon, Agim Ballvora

This PDF file contain the following information:

Supplementary Figure S1: Pearson product-moment correlations coefficient between the drought responses of two variables under drought condition.

Supplementary Figure S2. Association mapping for root volume (RV) traits under drought conditions.

Supplementary Figure S3: Association mapping for root surface area (RSA) traits under drought.

Supplementary Figure S4. Association mapping for the number of root forks (NRF) traits under drought.

Supplementary Figure S5. Association mapping for the number of root crossing (NRC) traits under drought.

Supplementary Figure S6: Chromosomal location of the associated SNPs to the root architectural traits of wheat under drought stress condition.

Supplementary Table S1. Annotation and ontology classification of candidate genes identified for root architectural traits in response to plasticity and drought stress.

Supplementary Table S2: Expression data of selected candidate genes in wheat within different tissues and development stages.

Supplementary Table S3: Expression data of selected candidate genes under 1 and 6 hours of drought stress.

Supplementary Figure S1: Pearson product-moment correlations coefficient between the drought responses of two variables under drought condition. The abbreviations indicate; total root length (TRL), number of root forks (NRF), root surface area (RSA), number of root tips (NRT), number of root crossing (NRC), root volume (RV), and root average diameter (RAD).

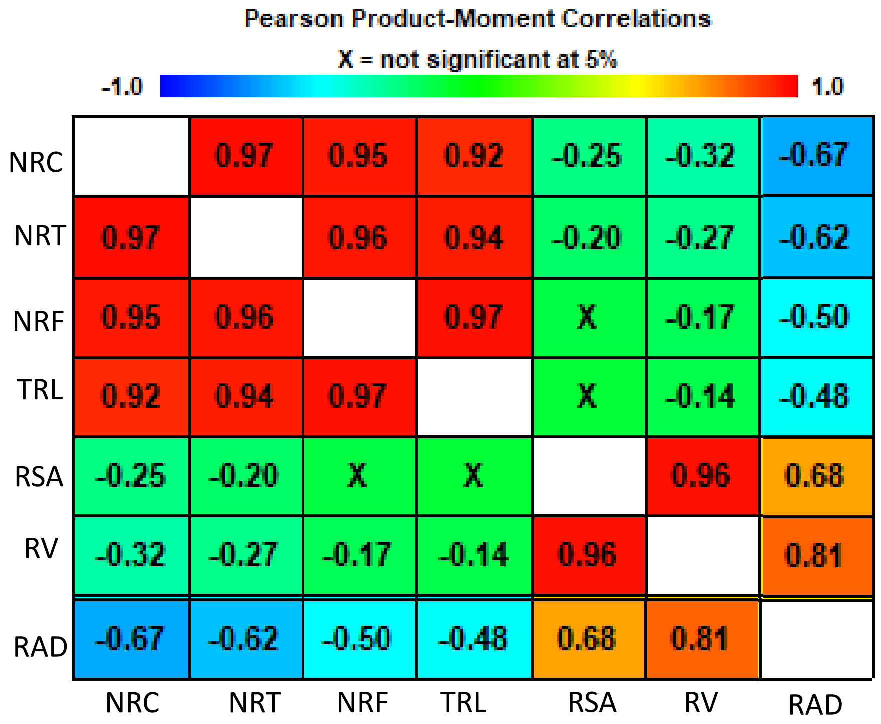

Supplementary Figure S2. Association mapping for root volume (RV) traits under drought conditions. (A) the histogram shows the frequency distribution of log-transformed data of RV traits and the blue and red color middle lines indicate the mean and median of the data set. (B) Quantile- Quantile plot of GWAS p values showing Y-axis: observed negative log 10(P-value) and X-axis expected negative log (p-value). (C) Rectangular Manhattan plot from association mapping of RV using a mixed linear model (MLM) considering the kinship and population structure, Y-axis: -$\log_{10}$ (*p*-value) and X-axis: the entire 21 chromosomes of the wheat genome**.** The red SNPs above the black line indicated the significant SNPs which passed the threshold level at *p*≤0.0001. The black SNPs above the dotted black line represented all the SNPs that did not reach the threshold level.

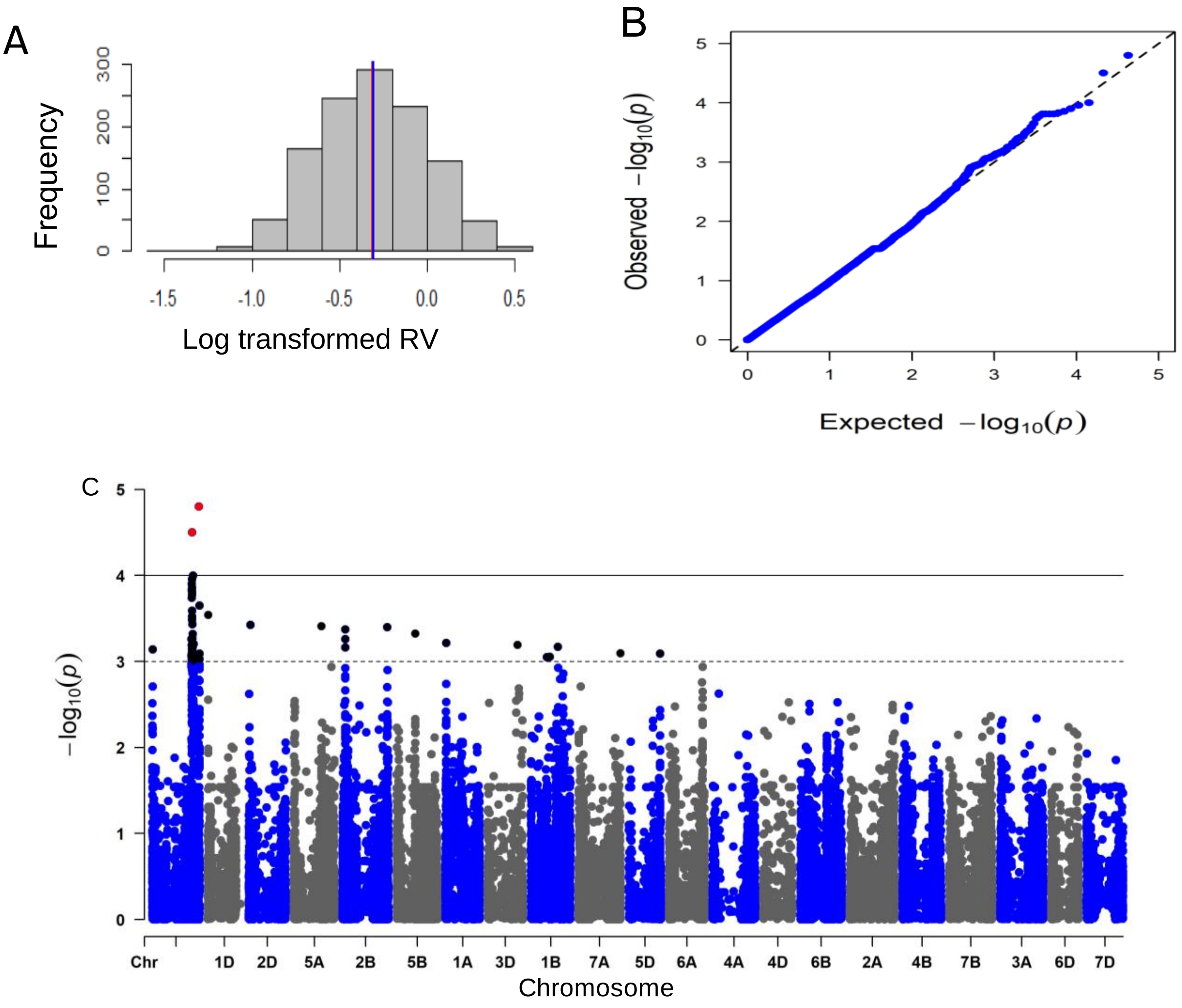

Supplementary Figure S3: Association mapping for root surface area (RSA) traits under drought. (A) the histogram shows the frequency distribution of log-transformed data of root fork (RF) and the blue and red color middle lines indicated the mean and median of the data set. (B) Quantile- Quantile plot of GWAS *p*-values showing Y-axis: observed -$\log_{10}$ (*p*-value) and X-axis expected -$\log_{10}$ (*p*-value). (C) Rectangular Manhattan plot from association mapping of RSA using a mixed linear model (MLM) considering the kinship and population structure, Y-axis: -$\log_{10}$ (*p*-value) and X-axis: the entire 21 chromosomes of the wheat genome**.** The red SNPs above the black line indicated the significant SNPs which passed the threshold level at *p*≤0.0001. The black SNPs above the dotted black line represented all the SNPs that did not reach the threshold level.

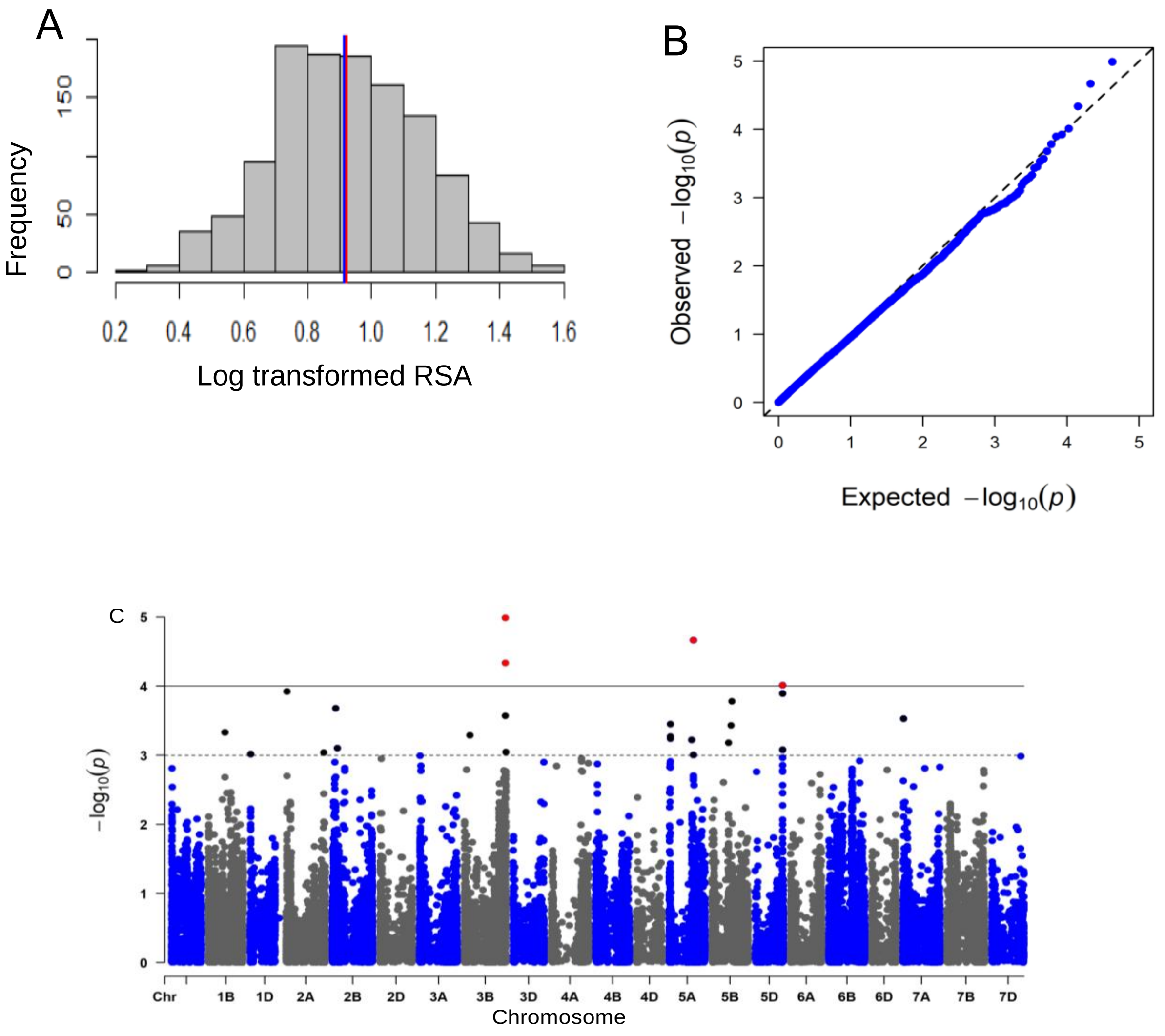

Supplementary Figure S4. Association mapping for the number of root forks (NRF) traits under drought. (A) The histogram shows the frequency distribution of log-transformed data of NRF and the blue and red color middle lines indicated the mean and median of the data set. (B) Quantile- Quantile plot of GWAS *p*-values showing Y-axis: observed -log 10 (*p*-value) and X-axis expected negative log (*p*-value). (C) Rectangular Manhattan plot from association mapping of NRF using a mixed linear model (MLM) considering the kinship and population structure, Y-axis: negative log10 (*p*-value) and X-axis: the entire 21 chromosomes of the wheat genome**.** The red SNPs above the black line indicated the significant SNPs which passed the threshold level at p≤0.0001. The black SNPs above the dotted black line represented all the SNPs that did not reach the threshold level.

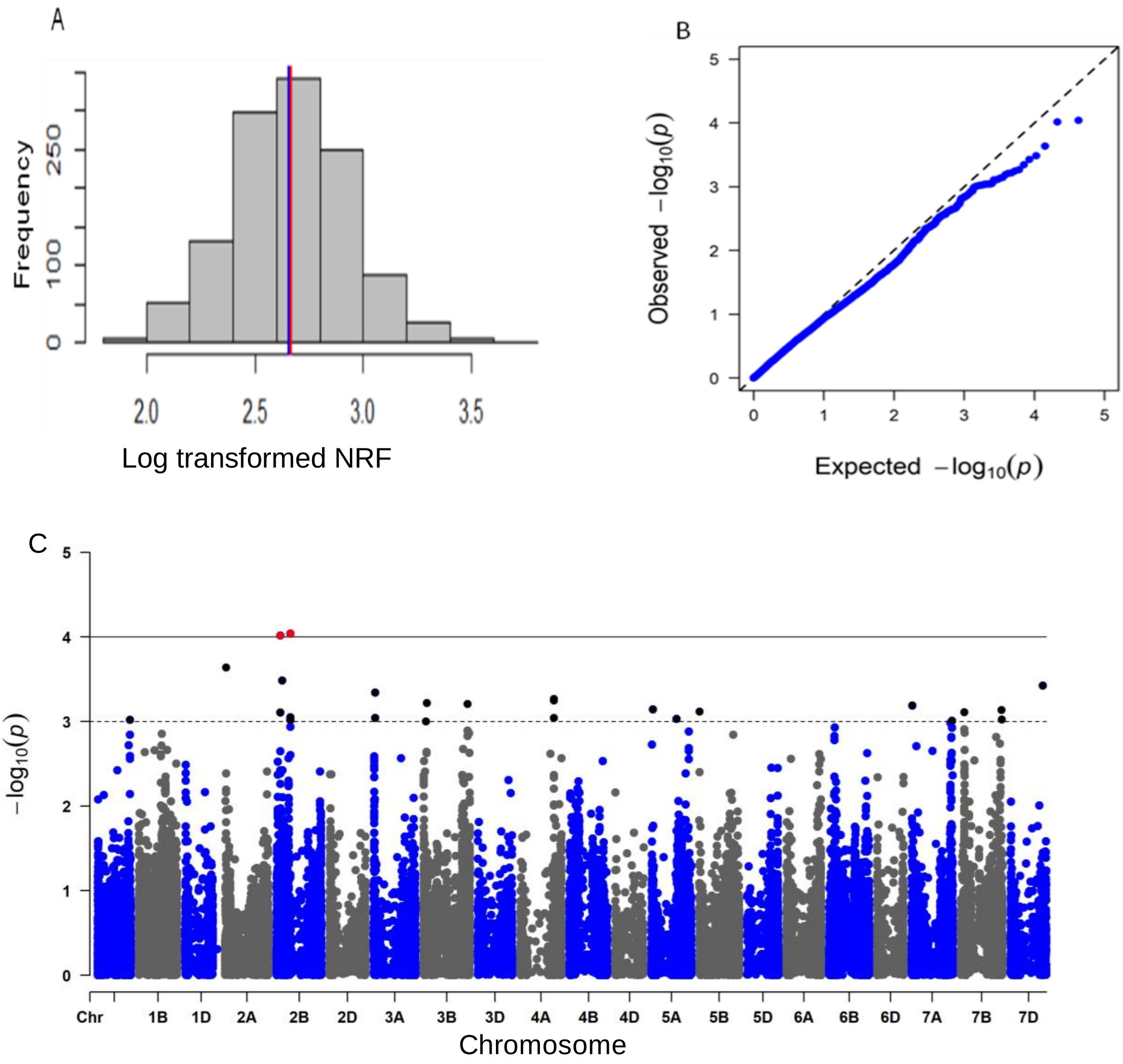

Supplementary Figure S5. Association mapping for the number of root crossing (NRC) traits under drought. (A)The histogram shows the frequency distribution of the log-transformed data number of root crossings (NRC). The blue and red color middle lines indicated the mean and median of the data set. (B) Quantile- Quantile plot of GWAS p values showing Y-axis: observed negative log 10(P-value) and X-axis expected negative log (*p*-value). (C) Rectangular

Manhattan plot from association mapping of RC using a mixed linear model (MLM) considering the kinship and population structure, Y-axis: negative log10 (*p*-value) and X-axis: the entire 21 chromosomes of the wheat genome**.** The red SNPs above the black line indicated the significant SNPs which passes the passes the threshold level at p≤0.0001. The black SNPs above the dotted black line represented all the SNPs that did not reach the threshold level.

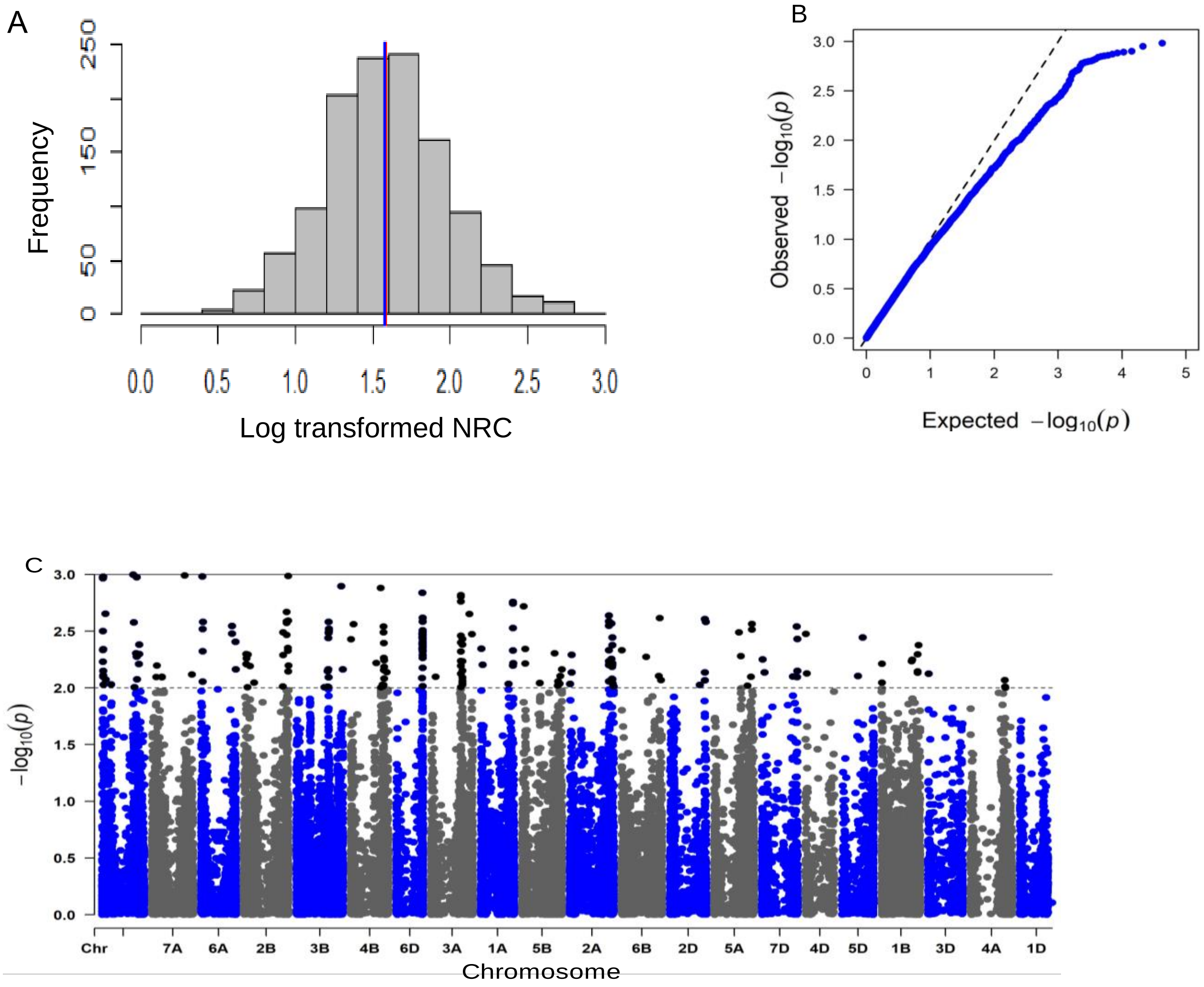

Supplementary Figure S6: Chromosomal location of the associated SNPs to the root architectural traits of wheat under drought stress condition. The name of the markers written on the left side of the chromosome and the right side of the chromosome indicated their position. The red-color markers indicated the significant SNPs markers on the chromosome.

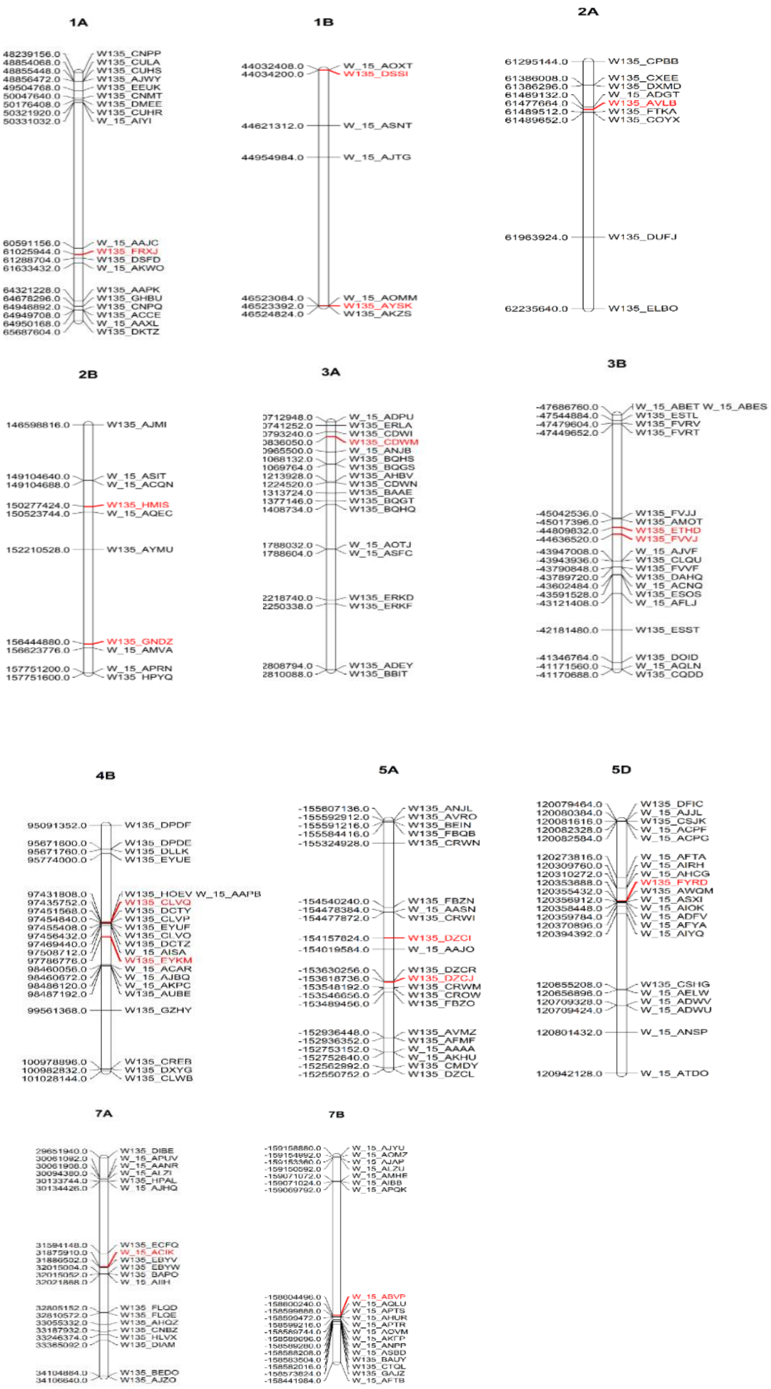

Supplementary Table S1. Annotation and ontology classification of candidate genes identified for root architectural traits in response to plasticity and drought stress.

Trait	Chr.	Gene ID	Gene size(bp)	Gene ontology annotation<br>Molecular function	 Biological function
TRL	1A(Block2)	TraesCS1A02G294000	3,703	abscisic acid binding- protein phosphatase inhibitor activity -signaling receptor activity(GO:0005488)	abscisic acid-activated signaling pathway -regulation of protein serine/threonine phosphatase activity(GO:0005488)
		TraesCS1A02G294500	3,731	potassium ion leak channel activity(GO:0016021), abscisic acid binding -protein phosphatase inhibitor activity -signaling receptor activity(GO:0031323)	potassium ion transmembrane transport -stabilization of membrane potential (GO:0016021), abscisic acid-activated signaling pathway -regulation of protein serine/threonine phosphatase activity (GO:0031323)
		TraesCS1A02G294100	2,178	abscisic acid binding-protein phosphatase inhibitor activity-signaling receptor activity(GO:0006464)	abscisic acid-activated signaling pathway-regulation of protein serine/threonine phosphatase activity(GO:0006464)
		TraesCS1A02G294600	1,359	hydrolase activity(GO:0006261), abscisic acid binding-protein phosphatase inhibitor activity-signaling receptor activity(GO:0050789)	DNA-dependent DNA replication -DNA repair (GO:0006261), abscisic acid-activated signaling pathway-regulation of protein serine/threonine phosphatase activity
		TraesCS1A02G294200	3,874	abscisic acid binding-protein phosphatase inhibitor activity-signaling receptor activity (GO:0050794), potassium ion leak channel activity (GO:0051179)	abscisic acid-activated signaling pathway-regulation of protein serine/threonine phosphatase activity(GO:0050794), stabilization of membrane potential(GO:0051179)
		TraesCS1A02G294300	4,020	potassium ion leak channel activity(GO:0065007)	potassium ion transmembrane transport -stabilization of membrane potential(GO:0065007)
		TraesCS1A02G294400	1,646	DNA binding-DNA-directed DNA polymerase activity- nucleotide binding (GO:0033554)	DNA replication- nucleic acid phosphodiester bond hydrolysis (GO:0033554)

TraesCS1A02G294700	1,178	RNA polymerase II regulatory region sequence-specific DNA binding (GO:0006351)	Cell fate specification-negative and positive regulation of transcription by RNA polymerase II (GO:0006351)
TraesCS1A02G294800	1,574	methyltransferase activity-RNA binding-tRNA (cytosine-5-)-methyltransferase activity(GO:0008168)	RNA methylation(GO:0008168)
TraesCS1A02G294900	2,570	potassium ion leak channel activity(GO:0065007)	potassium ion transmembrane transport -stabilization of membrane potential(GO:0065007)
TraesCS1A02G295100	1,591	potassium ion leak channel activity(GO:0006810), ion channel activity(GO:0019867)	potassium ion transmembrane transport -stabilization of membrane potential(GO:0006810), cation transport(GO:0019867)
TraesCS1A02G295200	7,552	hydrolase activity, hydrolyzing O-glycosyl compounds (GO:0016020)	hydrolase activity, hydrolyzing O-glycosyl compounds(GO:0016020)
TraesCS1A02G295300	2,911	potassium ion leak channel activity (GO:0005887), abscisic acid binding-signaling receptor activity (GO:0009738)	potassium ion transmembrane transport,stabilization of membrane potential(GO:0005887), abscisic acid-activated signaling pathway(GO:0009738), response to cold(GO:0009409)
TraesCS1A02G295500	261	specific DNA binding(GO:0008283), DNA-binding transcription activator activity, RNA polymerase II-specific (GO:0009888)	cellular response to hormone stimulus -positive regulation of cell differentiation (GO:0008283) , tissue development- cell fate specification (GO:0009888)
TraesCS1A02G295600	15,297	protein self-association -unfolded protein binding(GO:0009408)	response to heat -response to hydrogen peroxide-response to reactive oxygen species -response to salt stress(GO:0009408)
TraesCS1A02G295700	522	hydrolase activity, hydrolyzing O-glycosyl compounds(GO:0005886)	carbohydrate metabolic process(GO:0005886)
TraesCS1A02G295800	2,757	DNA binding-DNA topoisomerase activity (GO:0006139)	DNA topological change (GO:0006139)
TraesCS1A02G295900	1,410	DNA-binding transcription activator activity, RNA polymerase II-specific(GO:0032502)	cell fate specification(GO:0032502)

	TraesCS1A02G296000	438	abscisic acid binding-protein phosphatase inhibitor activity-signaling receptor activity (GO:0023052), potassium ion leak channel activity(GO:0016021)	abscisic acid-activated signaling pathway-regulation of protein serine/threonine phosphatase activity-signaling (GO:0023052), potassium ion transmembrane transport-stabilization of membrane potential(GO:0016021)
	TraesCS1A02G296400	687	potassium ion leak channel activity(GO:0016021)	potassium ion transmembrane transport-stabilization of membrane potential(GO:0016021),
1B(Block1)	TraesCS1B02G268800	4,656	water channel activity (GO:0009414)	response to water deprivation (GO:0009414)
	TraesCS1B02G268700	4,154	DNA-binding transcription activator activity, RNA polymerase II-specific(GO:0032502)	cell fate specification(GO:0032502)
	TraesCS1B02G269700	2,502	abscisic acid binding -signaling receptor activity (GO:0042221)	abscisic acid- regulation of protein serine/threonine phosphatase activity(GO:0042221)
	TraesCS1B02G269600	3,713	translation elongation factor activity-Elongation factor (GO:0003746) , phospholipase A1 activity (GO:0009695)	Protein biosynthesis (GO:0003746), jasmonic acid biosynthetic process-lipid metabolic process(GO:0009695)
	TraesCS1B02G270900	3,441	abscisic acid binding-protein phosphatase inhibitor activity-signaling receptor activity(GO:0050896)	abscisic acid-activated signaling pathway -regulation of protein serine/threonine phosphatase activity, response to stimulus (GO:0050896)
	TraesCS1B02G271700	5,901	abscisic acid binding-protein phosphatase inhibitor activity-signaling receptor activity (GO:0050790)	abscisic acid-activated signaling pathway -regulation of protein serine/threonine phosphatase activity(GO:0050790)
	TraesCS1B02G272300	999	abscisic acid binding -signaling (GO:0050794)	abscisic acid-regulation of protein serine/threonine phosphatase activity(GO:0050794)
	TraesCS1B02G272500	1,284	DNA binding-zinc ion binding(GO:0009723)	response to auxin-response to ethylene-response to gibberellin-response to ethylene (GO:0009723)
	TraesCS1B02G268900	927	abscisic acid binding-protein phosphatase inhibitor activity-signaling receptor activity(GO:0009725)	abscisic acid-activated signaling pathway-regulation of protein serine/threonine phosphatase activity-response to hormone (GO:0009725)

TraesCS1B02G269200	1,155	hydrolase activity, hydrolyzing O-glycosyl compounds(GO:0016020)	carbohydrate metabolic process(GO:0016020)
TraesCS1B02G269500	4,370	DNA helicase activity-single-stranded DNA binding(GO:0016787)	hydrolase activity (GO:0016787)
TraesCS1B02G269800	3,339	potassium ion leak channel activity(GO:0016021)	potassium ion transmembrane transport-stabilization of membrane potential (GO:0016021)
TraesCS1B02G269900	3,426	potassium ion leak channel activity(GO:0016021), sequence-specific DNA binding (GO:0009863)	stabilization of membrane potential (GO:0016021), hyper osmotic salinity-response to hormones(GO:0009863)
TraesCS1B02G270000	7,654	abscisic acid binding -signaling receptor activity(GO:0019222)	abscisic acid-regulation of protein serine/threonine phosphatase activity(GO:0019222)
TraesCS1B02G270300	2,889	potassium ion leak channel activity(GO:0016021)	potassium ion transmembrane transport-stabilization of membrane potential (GO:0016021)
TraesCS1B02G270100	5,416	hydrolase activity, hydrolyzing O-glycosyl compounds (GO:0005886)	carbohydrate metabolic process (GO:0005886)
TraesCS1B02G270300	2,889	potassium ion leak channel activity(GO:0016021)	potassium ion transmembrane transport-stabilization of membrane potential (GO:0016021)
TraesCS1B02G270200	1,313	potassium ion leak channel activity(GO:0016021), abscisic acid binding-signaling receptor activity (GO:0050896)	stabilization of membrane potential (GO:0016021), abscisic acid-activated signaling pathway-regulation of protein serine/threonine phosphatase activity-response to stimulus (GO:0050896)
TraesCS1B02G270400	4,077	potassium ion leak channel activity(GO:0065007)	potassium ion transmembrane transport-stabilization of membrane potential(GO:0065007)- tissue development (GO:0009888)
TraesCS1B02G270500	1,206	ATP binding(GO:0005524)	5-phosphoribose 1-diphosphate biosynthetic process- purine nucleotide biosynthetic process (GO:0005524)
TraesCS1B02G270600	6,014	NA	positive regulation of hydrolase activity (GO:0051345)
TraesCS1B02G270700	5,157	abscisic acid binding -signaling receptor activity (GO:0044267)	abscisic acid-regulation of protein serine/threonine phosphatase activity (GO:0044267)

TraesCS1B02G270800	3,493	protein self-association-unfolded protein binding(GO:0006950)	response to heat- response to reactive oxygen species-response to salt stress- response to stress (GO:0006950)
TraesCS1B02G271000	14,313	abscisic acid binding- signaling receptor activity(GO:0050789)	abscisic acid -regulation of protein serine/threonine phosphatase activity(GO:0050789)
TraesCS1B02G271100	7,518	abscisic acid binding -signaling receptor activity(GO:0050789)	abscisic acid -regulation of protein serine/threonine phosphatase activity(GO:0050789)
TraesCS1B02G271200	3,146	potassium ion leak channel activity(GO:0016021)	potassium ion transmembrane transport-stabilization of membrane potential- (GO:0016021)
TraesCS1B02G271300	4,924	abscisic acid binding -signaling receptor activity(GO:0009892)	abscisic acid -regulation of protein serine/threonine phosphatase activity(GO:0009892)
TraesCS1B02G271400	387	potassium ion leak channel activity(GO:0016021)	potassium ion transmembrane transport-stabilization of membrane potential (GO:0016021)
TraesCS1B02G271800	6,823	abscisic acid binding -signaling receptor activity (GO:0019222)	abscisic acid-regulation of protein serine/threonine phosphatase activity(GO:0019222)
TraesCS1B02G271900	1,832	potassium ion leak channel activity (GO:0065007)	potassium ion transmembrane transport-stabilization of membrane potential(GO:0065007)
TraesCS1B02G272200	4,753	potassium ion leak channel activity(GO:0016021)	potassium ion transmembrane transport-stabilization of membrane potential (GO:0016021)
TraesCS1B02G272600	2,336	abscisic acid binding-protein phosphatase inhibitor activity-signaling receptor activity (GO:0042221)	abscisic acid-activated signaling pathway-regulation of protein serine/threonine phosphatase activity (GO:0042221)
TraesCS1B02G272800	7,406	potassium ion leak channel activity(GO:0016021)	potassium ion transmembrane transport-stabilization of membrane potential (GO:0016021)
TraesCS1B02G272700	2,215	potassium ion leak channel activity(GO:0006810)	potassium ion transmembrane transport-stabilization of membrane potential-transport (GO:0006810)
TraesCS1B02G272400	2,297	potassium ion leak channel activity(GO:0016021)	potassium ion transmembrane transport-stabilization of membrane potential (GO:0016021)

2A(Block2)	TraesCS2A02G144600	243	RNA polymerase II regulatory region sequence-specific DNA binding(GO:0032502)	cell fate specification negative and positive regulation of transcription by RNA polymerase II-developmental process (GO:0032502)
	TraesCS2A02G144700	207	RNA polymerase II regulatory region sequence-specific DNA binding(GO:0031327)	cell fate specification negative and positive regulation of transcription by RNA polymerase II-developmental process(GO:0031327)
	TraesCS2A02G144800	3,488	translation elongation factor activity(GO:0003746)	Protein biosynthesis(GO:0003746)
	TraesCS2A02G145100	1,903	potassium ion leak channel activity(GO:0016021)	potassium ion transmembrane transport-stabilization of membrane potential (GO:0016021)
	TraesCS2A02G145200	5,318	hydrolase activity, hydrolyzing O-glycosyl compounds(GO:0016020)	carbohydrate metabolic process(GO:0016020)
2B(Block3)	TraesCS2B02G408800	8,275	abscisic acid binding -signaling receptor activity(GO:0032268)	abscisic acid-regulation of protein serine/threonine phosphatase activity(GO:0032268)
	TraesCS2B02G409100	2,072	amino acid transmembrane transporter activity- (GO:0015824)	amino acid transmembrane transport-proline transport (GO:0015824)
	TraesCS2B02G410400	4,291	abscisic acid binding-protein phosphatase inhibitor activity-signaling receptor activity(GO:0010033)	abscisic acid-activated signaling pathway -regulation of protein serine/threonine phosphatase activity(GO:0010033)
	TraesCS2B02G410600	8,832	chlorophyll binding (GO:0015979)	photosynthesis, light harvesting in photosystem I -response to light stimulus (GO:0015979)
	TraesCS2B02G409800	1,306	abscisic acid binding -signaling receptor activity-signal transduction (GO:0007165)	abscisic acid-regulation of protein serine/threonine phosphatase activity (GO:0007165)
	TraesCS2B02G410600	8,832	abscisic acid binding -signaling receptor activity-signal transduction (GO:0007165)	abscisic acid-regulation of protein serine/threonine phosphatase activity (GO:0007165)
	TraesCS2B02G410500	1,213	ubiquitin-protein transferase activity-zinc ion binding- (GO:0012501)	regulation of apoptotic process-programmed cell death (GO:0012501)

	TraesCS2B02G411500	1,188	ethylene binding-ethylene receptor activity (GO:0009873)	ethylene-activated signaling pathway (GO:0009873)
3A(Block3)	TraesCS3A02G039000	4,478	DNA binding-zinc ion binding(GO:0009733)	response to ethylene-response to gibberellin -response to auxin (GO:0009733)
	TraesCS3A02G039100	2,379	nitrate transmembrane transporter activity(GO:0010167) ,transporter activity (GO:0006826)	cellular response to nitrate- (GO:0010167) , transmembrane transport (GO:0006826)
	TraesCS3A02G039200	2,695	abscisic acid binding- -signaling receptor activity (GO:0007165), hydrolase activity (GO:0005886)	abscisic acid -regulation of protein serine/threonine phosphatase activity (GO:0007165) , carbohydrate metabolic process- plasma membrane (GO:0005886)
	TraesCS3A02G039800	1,623	potassium ion leak channel activity (GO:0016021)	potassium ion transmembrane transport-stabilization of membrane potential (GO:0016021)
	TraesCS3A02G039300	2,685	potassium ion leak channel activity (GO:0016021)	potassium ion transmembrane transport-stabilization of membrane potential (GO:0016021)
	TraesCS3A02G039400	3,624	potassium ion leak channel activity (GO:0016021)	potassium ion transmembrane transport-stabilization of membrane potential (GO:0016021)
	TraesCS3A02G039500	2,781	potassium ion leak channel activity (GO:0016021)	potassium ion transmembrane transport-stabilization of membrane potential (GO:0016021)
	TraesCS3A02G039600	1,885	transmembrane transporter activity-transporter activity (GO:0005783)	Transport (GO:0005783)
	TraesCS3A02G039700	6,584	abscisic acid binding-protein phosphatase inhibitor activity-signaling receptor activity(GO:0007165)	abscisic acid-activated signaling pathway-regulation of protein serine/threonine phosphatase activity- signal transduction (GO:0007165)
	TraesCS3A02G040100	3,151	potassium ion leak channel activity (GO:0016021)	potassium ion transmembrane transport-stabilization of membrane potential (GO:0016021)
4B(Block1)	TraesCS4B02G259900	2,400	potassium ion leak channel activity(GO:0065007)	potassium ion transmembrane transport-stabilization of membrane potential(GO:0065007)

		TraesCS4B02G260000	2,545	abscisic acid binding-protein phosphatase inhibitor activity-signaling receptor activity(GO:0050794)	abscisic acid-activated signaling pathway-regulation of protein serine/threonine phosphatase activity(GO:0050794)
		TraesCS4B02G260200	2,261	potassium ion leak channel activity (GO:0016021)	potassium ion transmembrane transport-stabilization of membrane potential (GO:0016021)
	5A(Block1)	TraesCS5A02G551600	4,721	potassium ion leak channel activity (GO:0006810)	potassium ion transmembrane transport-stabilization of membrane potential-transport (GO:0006810)
		TraesCS5A02G551900	387	abscisic acid binding- signaling receptor activity(GO:0008289), potassium ion leak channel activity (GO:0016021)	abscisic acid-activated signaling pathway -regulation of protein serine/threonine phosphatase activity(GO:0008289),stabilization of membrane potential(GO:0016021)
		TraesCS5A02G552600	4,373	abscisic acid binding -signaling receptor activity(GO:0048519)	abscisic acid-regulation of protein serine/threonine phosphatase activity(GO:0048519)
RAD	2A(Block2)	TraesCS2A02G438800	8,465	abscisic acid binding- signaling receptor activity(GO:0009737),	auxin-activated signaling pathway-transmembrane transport (GO:0009734)- xylem development(GO:0010089), abscisic acid-activated signaling pathway -regulation of protein serine/threonine phosphatase activity(GO:0009737)
		TraesCS2A02G438900	6,573	sodium-independent organic anion transmembrane transporter activity(GO:0006820), abscisic acid binding-protein phosphatase inhibitor activity-signaling receptor activity(GO:0010427)	cellular response to cold (GO:0070417), developmental process (GO:0051452), regulation of ion transmembrane transport (GO:0034765), abscisic acid-regulation of protein serine/threonine phosphatase activity(GO:0010427)
		TraesCS2A02G439300	2,540	abscisic acid binding-protein phosphatase inhibitor activity-signaling receptor activity(GO:0050794)	intracellular protein transport-retrograde transport, endosome to Golgi (GO:0012505), abscisic acid-activated signaling pathway -regulation of protein serine/threonine phosphatase activity(GO:0050794)
		TraesCS2A02G439500	1,849	hydrolase activity, hydrolyzing O-glycosyl compounds (GO:0046658)	carbohydrate metabolic process (GO:0046658)

	TraesCS2A02G439800	2,093	potassium ion leak channel activity, oxidoreductase activity(GO:0016021) , acting on paired donors, with incorporation or reduction of molecular oxygen (GO:0004497)	potassium ion transmembrane transport-stabilization of membrane potential (GO:0016021) ,
	TraesCS2A02G440100	4,638	Receptor (GO:0006623)	Golgi to endosome transport-Golgi to vacuole transport-post -mediated transport (GO:0006623), intracellular protein transport-retrograde transport(GO:0005770)
	TraesCS2A02G440300	318	protein self-association-unfolded protein binding(GO:0043933) , transmembrane transporter activity(GO:0005783)	response to heat-response to hydrogen peroxide-response to reactive oxygen species-response to salt stress(GO:0043933),Transport(GO:0005783)
	TraesCS2A02G440700	1,228	peroxidase activity-thioredoxin peroxidase activity(GO:0045454)	cell redox homeostasis-cellular response to oxidative stress(GO:0045454)
7A(no Block)	TraesCS7A02G062600	2,096	abscisic acid binding-protein phosphatase inhibitor activity-signaling receptor activity(GO:0023052)	cell wall organization-glucan catabolic process(GO:0005576), abscisic acid-regulation of protein serine/threonine phosphatase activity(GO:0023052)
	TraesCS7A02G062800	4,602	cation binding-hydrolase activity,(GO:0006112)	intracellular protein transport-photosystem II assembly-retrograde transport (GO:0005768) , glycogen biosynthetic process(GO:0006112)
	TraesCS7A02G063000	959	potassium ion leak channel activity (GO:0016021)	potassium ion transmembrane transport-stabilization of membrane potential (GO:0016021)
	TraesCS7A02G063700	6,709	potassium ion leak channel activity(GO:0000325)	potassium ion transmembrane transport-stabilization of membrane potential(GO:0000325)
	TraesCS7A02G064200	3,965	potassium ion leak channel activity (GO:0016021)	potassium ion transmembrane transport-stabilization of membrane potential(GO:0016021)
	TraesCS7A02G065600	1,959	cyclosporin A binding-peptidyl-prolyl cis-trans isomerase activity(GO:0070301)	cellular response to hydrogen peroxide, mitochondrial outer membrane permeabilization involved in programmed cell death,(GO:0070301)

RSA	3B(Block3)	TraesCS3B02G587900	9,933	ammonium transmembrane transporter activity-leak channel activity(GO:0072488)	ammonium transmembrane transport-cellular ion homeostasis(GO:0072488)
		TraesCS3B02G588000	4,328	abscisic acid binding -signaling receptor activity (GO:0050896)	abscisic acid-regulation of protein serine/threonine phosphatase activity-response to stimulus (GO:0050896)
		TraesCS3B02G588200	8,397	ATP binding-protein serine/threonine kinase activity(GO:0004672), abscisic acid binding-protein phosphatase inhibitor activity-signaling receptor activity(GO:0007165)	intracellular signal transduction- (GO:0004672), abscisic acid-regulation of protein serine/threonine phosphatase activity- signal transduction (GO:0007165)
		TraesCS3B02G588500	8,438	potassium ion leak channel activity (GO:0016021)	integral component of membrane (GO:0016021), potassium ion transmembrane transport-stabilization of membrane potential (GO:0016021)
		TraesCS3B02G588800	2,562	ammonium transmembrane transporter activity-leak channel activity(GO:0072488)	ammonium transmembrane transport-cellular ion homeostasis (GO:0072488)
		TraesCS3B02G588900	9,313	hydrolase activity, hydrolyzing O-glycosyl compounds (GO:0071944)	carbohydrate metabolic process (GO:0071944)
		TraesCS3B02G589100	7,214	ammonium transmembrane transporter activity-leak channel activity(GO:0072488)	ammonium transmembrane transport-cellular ion homeostasis(GO:0072488)
		TraesCS3B02G589300	5,221	ammonium transmembrane transporter activity-leak channel activity (GO:0072488)	ammonium transmembrane transport-cellular ion homeostasis-(GO:0072488)
		TraesCS3B02G589500	10,658	ATP binding-protein serine/threonine kinase activity (GO:0006468)	intracellular signal transduction-regulation of gene expression (GO:0006468)
		TraesCS3B02G589900	2,884	abscisic acid binding -signaling receptor activity (GO:0050794)	abscisic acid-regulation of protein serine/threonine phosphatase activity(GO:0050794)
		TraesCS3B02G590300	1,887	DNA-binding transcription factor activity-sequence-specific DNA binding (GO:0010014)	negative regulation of mitotic cell cycle-asymmetric cell division (GO:0010014)
		TraesCS3B02G590400	10,142	NA	intracellular protein transport-retrograde transport, endosome to Golgi(GO:0016482)

	5A(Block2)	TraesCS5A02G267400	1,242	abscisic acid binding- -signaling receptor activity(GO:0007154),selective channel activity-transmembrane signaling receptor activity(GO:0003008)	abscisic acid-regulation of protein serine/threonine phosphatase activity-cell communication (GO:0007154), -regulation of membrane potential-signal transduction (GO:0003008)
		TraesCS5A02G267200	2,563	protein serine/threonine kinase activity(GO:0032990), abscisic acid binding-protein phosphatase inhibitor activity-signaling receptor activity(GO:0044092)	establishment of cell polarity-intracellular signal transduction-cell part morphogenesis (GO:0032990), abscisic acid-regulation of protein serine/threonine phosphatase activity(GO:0044092)
		TraesCS5A02G267300	4,483	potassium ion leak channel activity(GO:0022857)	stabilization of membrane potential,transmembrane transporter activity (GO:0022857)
		TraesCS5A02G267800	417	abscisic acid binding -signaling receptor activity (GO:0050794)	abscisic acid-regulation of protein serine/threonine phosphatase activity(GO:0050794)
	5D(Block3)	TraesCS5D02G536600	857	potassium ion leak channel activity(GO:0016021)	potassium ion transmembrane transport-stabilization of membrane potential (GO:0016021)
NRF	2B(Block 1)	TraesCS2B02G104700	1,404	abscisic acid binding-signaling receptor activity(GO:0050896),hydrolase activity (GO:0048046)	abscisic acid-regulation of protein serine/threonine phosphatase activity-response to stimulus (GO:0050896), Hydrolase, apoplast (GO:0048046)
		TraesCS2B02G104800	3,323	amidase activity-glutaminyl-tRNA synthase (glutamine-hydrolyzing) activity(GO:0016879)	glutaminyl-tRNAGln biosynthesis via transamidation-mitochondrial translation(GO:0016879)
		TraesCS2B02G104900	1,085	abscisic acid binding-signaling receptor activity(GO:0007154)	abscisic acid-regulation of protein serine/threonine phosphatase activity-cell communication (GO:0007154)
		TraesCS2B02G105300	1,344	abscisic acid binding-protein phosphatase inhibitor activity-signaling receptor activity(GO:0038023)	abscisic acid binding-protein phosphatase inhibitor activity-signaling receptor activity-signaling receptor activity (GO:0038023)
		TraesCS2B02G237500	3,640	mRNA binding (GO:0003723)	posttranscriptional regulation of gene expression (GO:0003723)

TraesCS2B02G237600	1,386	translation regulator activity(GO:0048522), potassium ion leak channel activity(GO:0016021)	positive regulation of mitochondrial translation (GO:0048522),stabilization of membrane potential- (GO:0016021)
TraesCS2B02G237800	1,361	serine-type carboxypeptidase activity(GO:0008236)	proteolysis involved in cellular protein catabolic process-serine-type peptidase activity (GO:0008236)
TraesCS2B02G237900	6,652	threonine-type endopeptidase activity (GO:0000502)	protein catabolic process (GO:0000502),regulation of unidimensional cell growth (GO:0051510)
TraesCS2B02G238000	2,895	metalloendopeptidase activity(GO:0008233), RNA polymerase II activating transcription factor binding(GO:0000977)	inducible membrane protein ectodomain proteolysis-peptidase activity (GO:0008233), cell fate specification-negative and positive regulation of transcription by RNA polymerase II(GO:0000977)
TraesCS2B02G238100	467	protein self-association-unfolded protein binding(GO:0006950) , abscisic acid binding-protein phosphatase inhibitor activity-signaling receptor activity(GO:0050794)	response to heat-response to hydrogen peroxide-response to reactive oxygen species-response to salt stress-response to stress (GO:0006950) , abscisic acid-regulation of protein serine/threonine phosphatase activity(GO:0050794)
TraesCS2B02G238200	2,473	DNA-binding transcription activator activity, RNA polymerase II-specific(GO:0035295)	cell fate specification-negative and positive regulation of transcription by RNA polymerase II-tube development (GO:0035295)
TraesCS2B02G238300	3,329	argininosuccinate lyase activity(GO:0006526)	arginine biosynthetic process via ornithine(GO:0006526)
TraesCS2B02G238400	1,668	abscisic acid binding-signaling receptor activity(GO:0007165)	abscisic acid binding-protein phosphatase inhibitor activity- signal transduction (GO:0007165)
TraesCS2B02G238600	3,646	RNA polymerase II-specific(GO:0035295), abscisic acid binding-protein phosphatase inhibitor activity-signaling receptor activity(GO:0060089)	cell fate specification-negative and positive regulation of transcription by RNA polymerase II-tube development (GO:0035295),auxin transport (GO:0010540),regulation of meristem growth (GO:0010075), positive gravitropism (GO:0009958),response to blue light (GO:0009637), abscisic acid binding-protein phosphatase inhibitor activity (GO:0060089)

TraesCS2B02G238500	3,991	aminoacyl-tRNA hydrolase activity(GO:0004045)	aminoacyl-tRNA hydrolase activity (GO:0004045)
TraesCS2B02G238700	372	abscisic acid binding-signaling receptor activity(GO:0050896),	abscisic acid binding -response to stimulus (GO:0050896), intracellular protein transport-retrograde transport, (GO:0005829)
TraesCS2B02G238800	2,662	abscisic acid binding -signaling receptor activity- (GO:0019222)	abscisic acid-regulation of protein serine/threonine phosphatase activity- regulation of metabolic process(GO:0019222)
TraesCS2B02G238900	10,331	heat shock protein binding (GO:0005844), translation regulator activity(GO:0019898),	cellular response to unfolded protein (GO:0005844), positive regulation of mitochondrial translation- extrinsic component of membrane (GO:0019898)
TraesCS2B02G239000	4,064	potassium ion leak channel activity(GO:0016021), D-xylulose reductase activity-zinc ion binding(GO:0055114)	potassium ion transmembrane transport-stabilization of membrane potential(GO:0016021),oxidation-reduction process (GO:0055114)
TraesCS2B02G239200	4,508	DNA helicase activity(GO:0016787), terpene synthase activity-transferase activity(GO:0016114)	hydrolase activity (GO:0016787), terpenoid biosynthetic process (GO:0016114)
TraesCS2B02G239300	6,409	potassium ion leak channel activities(GO:0016021)	potassium ion transmembrane transport-stabilization of membrane potential (GO:0016021)
TraesCS2B02G239400	4,706	abscisic acid binding -signaling receptor activity(GO:0007165), hydrolase activity, (GO:0016020)	abscisic acid-regulation of protein serine/threonine phosphatase activity-signal transduction (GO:0007165)
TraesCS2B02G239500	6,550	RNA polymerase II regulatory region sequence-specific DNA binding (GO:0003700)	cell fate specification-negative and positive regulation of transcription by RNA polymerase II (GO:0003700)
TraesCS2B02G239600	918	DNA helicase activity-single-stranded DNA binding (GO:0003677),nucleosomal DNA binding(GO:0006342)	nucleosome positioning-regulation of transcription, DNA-templated-chromosome condensation(GO:0006342)

		TraesCS2B02G239700	1,073	RNA polymerase II regulatory region sequence-specific DNA binding (GO:0000790)	cell fate specification-negative and positive regulation of transcription by RNA polymerase II (GO:0000790)
		TraesCS2B02G240000	1,197	response to red light(GO:0010114), photosynthesis activity(GO:0009767)	photosystem II assembly(GO:0009543), photosynthetic electron transport chain(GO:0009767)
		TraesCS2B02G240100	3,242	acyloxyacyl hydrolase activity (GO:0044247),electron transporter -photosynthesis activity (GO:0009523)	photosynthetic electron transport in photosystem II-protein-chromophore linkage(GO:0009523)
		TraesCS2B02G240200	2,983	oxidoreductase activity, oxygen as acceptor(GO:0009523)	photosynthetic electron transport in photosystem II-protein(GO:0009523), intracellular protein transport-retrograde transport,(GO:0005829), acyloxyacyl hydrolase activity(GO:0044247)
NRT	1A(Block2)	TraesCS1A02G294000	3,703	DNA helicase activity(GO:0005488),DNA topoisomerase type I (GO:0044237)	DNA topological change(GO:0044237)
		TraesCS1A02G294100	2,178	potassium ion leak channel activity(GO:0016021)	potassium ion transmembrane transport-stabilization of membrane potential (GO:0016021)
		TraesCS1A02G294200	3,874	potassium ion leak channel activity(GO:0016021)	potassium ion transmembrane transport-stabilization of membrane potential(GO:0016021)
		TraesCS1A02G294300	4,020	abscisic acid binding- signaling receptor activity(GO:0016788)	abscisic acid-regulation of protein serine/threonine phosphatase activity-hydrolase activity(GO:0016788)
		TraesCS1A02G294400	1,646	potassium ion leak channel activity(GO:0016021),DNA-directed DNA polymerase activity (GO:0033554)	potassium ion transmembrane transport-stabilization of membrane potential (GO:0016021),cellular response to stress (GO:0033554)
		TraesCS1A02G294500	3,731	potassium ion leak channel activity(GO:0016021), translation regulator activity(GO:0048518), abscisic acid binding-signaling receptor activity (GO:0031323)	potassium ion transmembrane transport-stabilization of membrane potential (GO:0016021),positive regulation of biological process (GO:0048518), abscisic acid-regulation of protein serine/threonine phosphatase activity(GO:0031323)

TraesCS1A02G294600	1,359	potassium ion leak channel activity(GO:0016021), abscisic acid binding-protein phosphatase inhibitor activity-signaling receptor activity(GO:0050789), hydrolase activity (GO:0006261)	potassium ion transmembrane transport-stabilization of membrane potential (GO:0016021), abscisic acid-activated signaling pathway-regulation of protein serine/threonine phosphatase activity-regulation of biological process (GO:0050789),
TraesCS1A02G294700	1,178	RNA polymerase II regulatory region sequence-specific DNA binding(GO:0045892)	cell fate specification-negative and positive regulation of transcription by RNA polymerase II(GO:0045892),
TraesCS1A02G294800	1,574	potassium ion leak channel activity(GO:0016021), DNA helicase activity (GO:0016787), abscisic acid binding- -signaling receptor activity(GO:0006464)	potassium ion transmembrane transport-stabilization of membrane potential (GO:0016021),hydrolase activity (GO:0016787),abscisic acid-regulation of protein serine/threonine phosphatase activity (GO:0006464)
TraesCS1A02G294900	2,570	potassium ion leak channel activity(GO:0065007)	potassium ion transmembrane transport-stabilization of membrane potential- (GO:0065007)
TraesCS1A02G29500	1,549	abscisic acid binding-signaling receptor activity(GO:0023052), protein self-association (GO:0006950), potassium ion leak channel activity(GO:0016021)	abscisic acid -regulation of protein serine/threonine phosphatase activity- signaling (GO:0023052),response to heat- response to reactive oxygen species-response to salt stress-response to stress (GO:0006950),stabilization of membrane potential (GO:0016021)
TraesCS1A02G295100	1,591	potassium ion leak channel activity(GO:0006810), abscisic acid binding-signaling receptor activity (GO:0050789)	stabilization of membrane potential-transport (GO:0006810), abscisic acid -regulation of protein serine/threonine phosphatase activity-regulation of biological process (GO:0050789)
TraesCS1A02G295200	7,552	DNA helicase activity-single-stranded DNA binding(GO:0016787)	hydrolase activity (GO:0016787)
TraesCS1A02G295300	2,911	potassium ion leak channel activity (GO:0005887), abscisic acid binding -signaling receptor activity (GO:0009738) , (GO:0009409)	stabilization of membrane potential- (GO:0005887), regulation of jasmonic acid mediated signaling pathway(GO:0009867), abscisic acid-regulation of protein serine/threonine phosphatase

			activity(GO:0009738), cellular response to light stimulus (GO:0009581), response to cold -Transport(GO:0009409)
TraesCS1A02G295600	15,297	hydrolase activity, hydrolyzing O-glycosyl compounds(GO:0005886), potassium ion leak channel activity(GO:0005774), potassium ion leak channel activity(GO:0065007)	intracellular protein transport-retrograde transport, (GO:0012505), potassium ion transmembrane transport-stabilization of membrane potential(GO:0005774) ,response to high light intensity (GO:0009644), potassium ion transmembrane transport-stabilization of membrane potential-biological regulation (GO:0065007),response to heat-response to reactive oxygen species-response to salt stress-(GO:0042542)
TraesCS1A02G295800	2,757	nucleic acid binding(GO:0010162), potassium ion leak channel activity	negative regulation of transcription, DNA-templated-seed dormancy process(GO:0010162), potassium ion transmembrane transport-stabilization of membrane potential(GO:0016021)
TraesCS1A02G295900	1,410	abscisic acid-activated signaling pathway-regulation of protein serine/threonine phosphatase activity(GO:0050794),RNA polymerase II regulatory region sequence-specific DNA binding(GO:0032502)	abscisic acid binding-protein phosphatase inhibitor activity-signaling receptor activity (GO:0050794), cell fate specification-negative and positive regulation of transcription by RNA polymerase II(GO:0032502), retrograde transport, (GO:0012505)
TraesCS1A02G296000	438	DNA helicase activity-single-stranded DNA binding(GO:0016787), abscisic acid-activated signaling pathway-regulation of protein serine/threonine phosphatase activity(GO:0023052), potassium ion leak channel activity (GO:0005773)	hydrolase activity (GO:0016787), abscisic acid binding- signaling receptor activity-signaling (GO:0023052), potassium ion transmembrane transport-stabilization of membrane potential(GO:0005773)
TraesCS1A02G296200	4,331	potassium ion leak channel activity(GO:0005215)	potassium ion transmembrane transport-stabilization of membrane potential- transporter activity (GO:0005215)
TraesCS1A02G296400	687	potassium ion leak channel activity(GO:0016021)	potassium ion transmembrane transport-stabilization of membrane potential integral component of membrane

2B	TraesCS2B02G407500	5,821	specific DNA binding-(GO:0009630)-hydrolase activity(GO:0005886)	cell fate specification-gravitropism(GO:0009630)- carbohydrate metabolic process(GO:0005886)
	TraesCS2B02G407600	1,755	RNA polymerase II regulatory region sequence-specific DNA binding(GO:0030154)	cell fate specification-negative and positive regulation of transcription by RNA polymerase II-cell differentiation (GO:0030154)
	TraesCS2B02G407700	2,483	potassium ion leak channel activity(GO:0016021),abscisic acid binding -signaling receptor activity(GO:0044267)	potassium ion transmembrane transport-stabilization of membrane potential (GO:0016021) abscisic acid -regulation of protein serine/threonine phosphatase activity(GO:0044267)
	TraesCS2B02G407800	1,604	potassium ion leak channel activity(GO:0016021)	potassium ion transmembrane transport-stabilization of membrane potential (GO:0016021)
	TraesCS2B02G407900	4,964	nucleic acid binding(GO:0048316), transmembrane transporter activity(GO:0034755)	negative regulation of transcription, DNA-templated-seed dormancy process-seed development (GO:0048316), transmembrane transport(GO:0034755)
	TraesCS2B02G408000	4,686	nucleic acid binding(GO:0048316), transmembrane transporter activity(GO:0034755)	transmembrane transport(GO:0034755)
	TraesCS2B02G408100	663	abscisic acid binding -signaling receptor activity(GO:0050794), protein self-association-unfolded protein binding(GO:0006950), hydrolase activity (GO:0005886)	abscisic acid-activated signaling pathway-regulation of protein serine/threonine phosphatase activity(GO:0050794),-response to heat-response to hydrogen peroxide-response to reactive oxygen species-response to salt stress- response to stress (GO:0006950),
	TraesCS2B02G408200	264	potassium ion leak channel activity(GO:0065008), abscisic acid binding-signaling receptor activity(GO:0048519), protein self-association-unfolded protein binding(GO:0006950)	stabilization of membrane potential(GO:0065008) , abscisic acid-activated signaling pathway-regulation of protein serine/threonine phosphatase activity(GO:0048519),response to heat-response to hydrogen peroxide-response to reactive oxygen species-response to salt stress-response to stress (GO:0006950)

	TraesCS2B02G408300	15,064	potassium ion leak channel activity-(GO:0065007), ATP binding-protein serine/threonine kinase activity(GO:0004672)	potassium ion transmembrane transport-stabilization of membrane potential-biological regulation (GO:0065007), intracellular signal transduction-regulation of gene expression- protein kinase activity (GO:0004672)
3A(Block3)	TraesCS3A02G038700	11,894	potassium ion leak channel activity(GO:0016021)	stabilization of membrane potential (GO:0016021), regulation of hydrogen peroxide (GO:0010310), salicylic acid mediated signaling pathway (GO:0009862), jasmonic acid mediated signaling pathway (GO:0009867)
	TraesCS3A02G038900	1,754	transporting ATP synthase activity, rotational mechanism(GO:0043531), D-xylulose reductase activity(GO:0055114)	ATP synthesis coupled proton transport(GO:0043531), - oxidation-reduction process (GO:0055114)
	TraesCS3A02G039100	2,379	potassium ion leak channel activity (GO:0016021), abscisic acid binding - signaling receptor activity(GO:0007165) , ATP binding-protein serine/threonine kinase activity(GO:0004674)	potassium ion transmembrane transport-stabilization of membrane potential (GO:0016021), abscisic acid-regulation of protein serine/threonine phosphatase activity (GO:0007165) , intracellular signal transduction-regulation of gene expression(GO:0004674)
	TraesCS3A02G039200	2,695	abscisic acid binding -signaling receptor activity(GO:0007165), potassium ion leak channel activity (GO:0016021)	abscisic acid-regulation of protein serine/threonine phosphatase activity-signal transduction (GO:0007165), potassium ion transmembrane transport-stabilization of membrane potential (GO:0016021)
	TraesCS3A02G039300	2,685	potassium ion leak channel activity (GO:0016021),protein serine/threonine kinase activity (GO:0004674)	potassium ion transmembrane transport-stabilization of membrane potential (GO:0016021), intracellular signal transduction-regulation of gene expression (GO:0004674)
	TraesCS3A02G039400	3,624	potassium ion leak channel activity (GO:0016021),protein serine/threonine kinase activity (GO:0004674)	potassium ion transmembrane transport-stabilization of membrane potential(GO:0016021), intracellular signal transduction-regulation of gene expression (GO:0004674)

	TraesCS3A02G039500	2,781	potassium ion leak channel activity (GO:0016021),protein serine/threonine kinase activity (GO:0004674)	potassium ion transmembrane transport-stabilization of membrane potential (GO:0016021), intracellular signal transduction (GO:0004674)
	TraesCS3A02G039600	1,885	potassium ion leak channel activity (GO:0016021),	potassium ion transmembrane transport-stabilization of membrane potential (GO:0016021), oxidation-reduction process (GO:0055114)
	TraesCS3A02G039700	6,584	abscisic acid binding-protein phosphatase inhibitor activity-signaling receptor activity	abscisic acid-activated signaling pathway-regulation of protein serine/threonine phosphatase activity-signal transduction (GO:0007165)
4B(Block2)	TraesCS4B02G259500	5,196	ATP binding-magnesium ion binding-ribose phosphate diphosphokinase activity(GO:0005524)	nucleoside metabolic process-nucleotide biosynthetic process-purine nucleotide biosynthetic process(GO:0005524)
	TraesCS4B02G259700	5,543	transferase activity, transferring alkyl or aryl (other than methyl) groups(GO:0005737)	isoprenoid biosynthetic process(GO:0005737), carbonate dehydratase activity-zinc ion binding(GO:0006730)
	TraesCS4B02G259800	3,000	potassium ion leak channel activity-biological regulation (GO:0065007), abscisic acid binding -signaling receptor activity- (GO:0051716), transmembrane transporter (GO:0015931)	potassium ion transmembrane transport-stabilization of membrane potential (GO:0065007), abscisic acid -regulation of protein serine/threonine phosphatase activity- cellular response to stimulus (GO:0051716)
	TraesCS4B02G259900	2,400	potassium ion leak channel activity(GO:0065007)	potassium ion transmembrane transport-stabilization of membrane potential(GO:0065007)
	TraesCS4B02G260000	2,545	serine-type peptidase activity(GO:0006626), abscisic acid binding -signaling receptor activity(GO:0023052)	protein targeting to mitochondrion (GO:0006626), abscisic acid-regulation of protein serine/threonine phosphatase activity(GO:0023052)
	TraesCS4B02G260100	3,877	hydrolase activity, hydrolyzing O-glycosyl compounds (GO:0005886)	carbohydrate metabolic process (GO:0005886)
	TraesCS4B02G260300	4,482	hydrolase activit,(GO:0009691), ATP binding-protein kinase	cytokinin biosynthetic process (GO:0009691), plasmodesma (GO:0009506) , intracellular protein

			activity(GO:0009506) ,potassium ion leak channel activity (GO:0005774)	transport (GO:0005794),stabilization of membrane potential (GO:0005774)
	TraesCS4B02G260600	2,393	hydrolase activityGO:0005886), abscisic acid binding-signaling receptor activity(GO:0050789)	abscisic acid-activated signaling pathway-regulation of protein serine/threonine phosphatase activity-regulation of biological process (GO:0050789)
	TraesCS4B02G260400	6,220	potassium ion leak channel activity(GO:0034220)	potassium ion transmembrane transport-stabilization of membrane potential-ion transmembrane transport (GO:0034220)
	TraesCS4B02G260500	5,957	hydrolase activity(GO:0005886), abscisic acid binding -signaling receptor activity (GO:0044267)	carbohydrate metabolic process(GO:0005886), abscisic acid-activated signaling pathway-regulation of protein serine/threonine phosphatase activity(GO:0044267)
	TraesCS4B02G260700	825	abscisic acid binding-protein phosphatase inhibitor activity-signaling receptor activity(GO:0007165)	abscisic acid-activated signaling pathway-regulation of protein serine/threonine phosphatase activity,signal transduction (GO:0007165)
	TraesCS4B02G260800	6,172	protein self-association-unfolded protein binding(GO:0044085), RNA polymerase II regulatory region sequence-specific DNA binding(GO:0009888)	response to heat-response to hydrogen peroxide-response to reactive oxygen species-response to salt stress(GO:0044085), cell fate specification-negative and positive regulation of transcription by RNA polymerase II- tissue development (GO:0009888)
	TraesCS4B02G260900	1,736	DNA helicase activity-single-stranded DNA binding (GO:0016043), abscisic acid binding-protein phosphatase inhibitor activity-signaling receptor activity(GO:0050794)	abscisic acid-activated signaling pathway-regulation of protein serine/threonine phosphatase activity-regulation of cellular process (GO:0050794)
	TraesCS4B02G261000	1,479	potassium ion leak channel activity(GO:0016021), abscisic acid binding-protein phosphatase inhibitor activity-signaling receptor activity(GO:0009725)	potassium ion transmembrane transport-stabilization of membrane potential (GO:0016021), abscisic acid-activated signaling pathway-regulation of protein serine/threonine phosphatase activity-response to hormone (GO:0009725)
5A(Block1)	TraesCS5A02G551300	2,788	DNA helicase activity-single-stranded DNA binding(GO:0003824)	catalytic activity (GO:0003824)

TraesCS5A02G551400	3,269	potassium ion leak channel activity(GO:0016021), abscisic acid binding - signaling receptor activity(GO:0007165)	potassium ion transmembrane transport-stabilization of membrane potential (GO:0016021), abscisic acid-regulation of protein serine/threonine phosphatase activity-signal transduction (GO:0007165)
TraesCS5A02G551500	3,246	DNA-binding transcription activator activity, RNA polymerase II-specific(GO:0032502), abscisic acid binding-signaling receptor activity(GO:0007165)- potassium ion leak channel activity(GO:0016021),	cell fate specification-negative and positive regulation of transcription by RNA polymerase II-developmental process (GO:0032502), abscisic acid-regulation of protein serine/threonine phosphatase activity-signal transduction (GO:0007165)- potassium ion transmembrane transport-stabilization of membrane potential (GO:0016021),hydrolase activity (GO:0016787)
TraesCS5A02G551600	4,721	abscisic acid binding- signaling receptor activity(GO:0007165), potassium ion leak channel activity(GO:0006810)	abscisic acid-activated signaling pathway-regulation of protein serine/threonine phosphatase activity-signal transduction (GO:0007165), potassium ion transmembrane transport-stabilization of membrane potential(GO:0006810)
TraesCS5A02G551800	1,881	potassium ion leak channel activity(GO:0016021), abscisic acid binding-signaling receptor activity(GO:0050789)	potassium ion transmembrane transport-stabilization of membrane potential (GO:0016021), abscisic acid-activated signaling pathway-regulation of protein serine/threonine phosphatase activity-regulation of biological process (GO:0050789)
TraesCS5A02G551900		growth factor activity(GO:0008284), protein self-association-unfolded protein binding(GO:0006950)	regulation of root meristem growth- positive regulation of cell population proliferation (GO:0008284), response to heat-response to hydrogen peroxide-response to reactive oxygen species-response to salt stress-response to stress (GO:0006950)
TraesCS5A02G552500	708	potassium ion leak channel activity(GO:0016021)	potassium ion transmembrane transport-stabilization of membrane potential- (GO:0016021), regulation of cell communication (GO:0010646)

		TraesCS5A02G552600	4,373	potassium ion leak channel activity(GO:0016021), abscisic acid binding-signaling receptor activity(GO:0048519)	potassium ion transmembrane transport-stabilization of membrane potential (GO:0016021), abscisic acid-activated signaling pathway-regulation of protein serine/threonine phosphatase activity (GO:0048519)
		TraesCS5A02G552700	8,033	potassium ion leak channel activity(GO:0016021),	potassium ion transmembrane transport-stabilization of membrane potential (GO:0016021),
RV	3B(Block1)	TraesCS3B02G453100	6,031	potassium ion leak channel activity(GO:0016021	potassium ion transmembrane transport-stabilization of membrane potential (GO:0016021),
		TraesCS3B02G453300	1,314	abscisic acid binding- signaling receptor activity(GO:0009737) ,protein self-association-unfolded protein binding (GO:0009651)	,cell differentiation (GO:0030154), abscisic acid-activated signaling pathway-regulation of protein serine/threonine phosphatase activity (GO:0009737), response to hormone (auxin, gibberellin)(GO:0009723), response to heat- response to reactive oxygen species (GO:0009651)
		TraesCS3B02G453400	1,550	abscisic acid binding-protein phosphatase inhibitor activity-signaling receptor activity(GO:0070887)	abscisic acid-activated signaling pathway-regulation of protein serine/threonine phosphatase activity(GO:0070887)
		TraesCS3B02G453700	3,957	5'-flap endonuclease activity-crossover junction endodeoxyribonuclease activity(GO:0090305)	double-strand break repair via homologous recombinationnucleic acid phosphodiester bond hydrolysis (GO:0090305)
		TraesCS3B02G453200	1,840	hydrolase activity(GO:0016020), abscisic acid binding-protein phosphatase inhibitor activity-signaling receptor activity(GO:0050896)	carbohydrate metabolic process(GO:0016020), abscisic acid-activated signaling pathway-regulation of protein serine/threonine phosphatase activity-response to stimulus (GO:0050896)
		TraesCS3B02G453500	3,245	peroxidase activity-thioredoxin peroxidase activity(GO:0045454), potassium ion leak channel activity(GO:0016021)	anatomical structure development (GO:0048856), cell redox homeostasis (GO:0045454), potassium ion transmembrane transport-stabilization of membrane potential (GO:0016021)
		TraesCS3B02G453600	1,248	peroxidase activity-thioredoxin peroxidase activity(GO:0045454)	response to light intensity (GO:0009642),cell redox homeostasis (GO:0045454)

	TraesCS3B02G453800	1,074	DNA-binding transcription activator activity, RNA polymerase II-specific(GO:0009888), potassium ion leak channel activity(GO:0016021)	cell fate specification-tissue development (GO:0009888),cell population proliferation (GO:0008283), stabilization of membrane potential (GO:0016021)
	TraesCS3B02G454000	9,123	potassium ion leak channel activity(GO:0034220), hydrolase activity(GO:0005886)	stabilization of membrane potential (GO:0034220), transmembrane transport(GO:0006857), carbohydrate metabolic process(GO:0005886)
	TraesCS3B02G454100	4,234	potassium ion leak channel activity(GO:0034220), potassium ion leak channel activity(GO:0016021)	stabilization of membrane potential (GO:0034220), transmembrane transport(GO:0006857), potassium ion transmembrane transport-stabilization of membrane potential (GO:0016021)
	TraesCS3B02G454190	7,498	abscisic acid binding- signaling receptor activity (GO:0050794), DNA helicase activity-single-stranded DNA binding(GO:0009987), hydrolase activity (GO:0016020)	abscisic acid -regulation of protein serine/threonine phosphatase activity (GO:0050794), anatomical structure development (GO:0048856), developmental process (GO:0032502), carbohydrate metabolic process(GO:0016020)
	TraesCS3B02G454200	1,767	5'-flap endonuclease activity-crossover junction endodeoxyribonuclease activity(GO:0004519)	leaf vascular tissue pattern formation (GO:0010305), phloem or xylem histogenesis (GO:0010087) ,cotyledon vascular tissue pattern formation (GO:0010588)
	TraesCS3B02G454300	6,301	protein self-association-unfolded protein binding(GO:0009651), DNA helicase activity-single-stranded DNA binding(GO:0016887)	response to heat-response to hydrogen peroxide-response to reactive oxygen species-response to salt stress-response to salt stress (GO:0009651), ATPase activity (GO:0016887)
	TraesCS3B02G454400	6,396	protein self-association-unfolded protein binding(GO:0009651)	response to heat-response to hydrogen peroxide-response to reactive oxygen species-response to salt stress(GO:0009651)
3B(Block3)	TraesCS3B02G588200	8,397	abscisic acid binding-protein phosphatase inhibitor activity-signaling receptor activity(GO:0007165)	abscisic acid-activated signaling pathway-regulation of protein serine/threonine phosphatase activity-signal transduction (GO:0007165)

TraesCS3B02G588300	2,559	potassium ion leak channel activity(GO:0016021) , G protein-coupled photoreceptor activity (GO:0009581)	potassium ion transmembrane transport-stabilization of membrane potential (GO:0016021),plant-type hypersensitive response (GO:0009626), cellular response to light stimulus (GO:0009581)
TraesCS3B02G588400	3,869	potassium ion leak channel activity(GO:0016021)	potassium ion transmembrane transport-stabilization of membrane potential (GO:0016021)
TraesCS3B02G588600	885	potassium ion leak channel activity(GO:0016021)	potassium ion transmembrane transport-stabilization of membrane potential (GO:0016021)
TraesCS3B02G588500	8,438	potassium ion leak channel activity(GO:0016021)	potassium ion transmembrane transport-stabilization of membrane potential (GO:0016021)
TraesCS3B02G588700	9,254	potassium ion leak channel activity(GO:0016021)	potassium ion transmembrane transport-stabilization of membrane potential (GO:0016021)
TraesCS3B02G588800	2,562	potassium ion leak channel activity(GO:0016021)	potassium ion transmembrane transport-stabilization of membrane potential (GO:0016021)
TraesCS3B02G588900	9,313	DNA helicase activity- (GO:0016787),, potassium ion leak channel activity(GO:0016021),	hydrolase activity (GO:0016787), potassium ion transmembrane transport-stabilization of membrane potential (GO:0016021)
TraesCS3B02G589000	7,383	potassium ion leak channel activity(GO:0016021)	potassium ion transmembrane transport-stabilization of membrane potential (GO:0016021)
TraesCS3B02G589400	1,143	potassium ion leak channel activity(GO:0016021)	potassium ion transmembrane transport-stabilization of membrane potential (GO:0016021)
TraesCS3B02G589500	10,658	potassium ion leak channel activity(GO:0016021)	potassium ion transmembrane transport-stabilization of membrane potential (GO:0016021)
TraesCS3B02G589600	5,350	potassium ion leak channel activity(GO:0016021)	potassium ion transmembrane transport-stabilization of membrane potential (GO:0016021)
TraesCS3B02G589700	11,881	potassium ion leak channel activity(GO:0016021)	potassium ion transmembrane transport-stabilization of membrane potential (GO:0016021)

TraesCS3B02G589900	2,884	potassium ion leak channel activity(GO:0016021)	potassium ion transmembrane transport-stabilization of membrane potential (GO:0016021)
TraesCS3B02G590100	1,326	potassium ion leak channel activity(GO:0016021)	potassium ion transmembrane transport-stabilization of membrane potential (GO:0016021)
TraesCS3B02G590400	10,142	hydrolase activity,(GO:0016020)	carbohydrate metabolic process(GO:0016020)
TraesCS3B02G590600	16,150	potassium ion leak channel activity(GO:0016021)	potassium ion transmembrane transport-stabilization of membrane potential (GO:0016021)
TraesCS3B02G590500	3,455	potassium ion leak channel activity(GO:0016021)	potassium ion transmembrane transport-stabilization of membrane potential (GO:0016021)

Candidate genes are located in linkage disequilibrium (LD) block embedded the significant single nucleotide polymorphism (SNP) markers. The significant SNPs which does not belong to an LD block, a 1Mbp window on either side of significant SNP was considered to search putative candidate genes. The available gene annotation and gene ontology(GO) was obtained from the wheat @URGI database(Alaux *et al*., 2018b). Abbreviation: **Car**, chromosome; TRL, total root length; RSA, root surface area; RAD, root average diameter; RV, root volume; NRT, number of root tips; NRF, number of root forks; and NA, not available.

Supplementary Table S2: Expression data of selected candidate genes in wheat within different tissues and development stages.

Genes	flag_leaf_1	flag_leaf_8	grain_10	leaf_105	root_apical_meristem_5	roots_10	roots_15	roots_20	roots_25	roots_35	roots_40	second_leaf_15	shoot_apical_meristem_5	shoots_25	spike_20
TraesCS1A02G295000	0.000	0.000	0.000	0.000	0.000	0.000	0.000	0.000	0.000	0.000	0.000	0.000	0.000	0.000	0.000
TraesCS1A02G295400	0.000	1.743	0.000	1.281	6.355	5.246	5.502	5.226	5.260	5.721	6.004	0.652	5.634	6.213	7.491
TraesCS1A02G296200	0.186	1.638	3.630	2.580	3.895	2.715	2.992	3.523	3.508	3.116	2.972	1.760	3.253	2.448	2.075
TraesCS1A02G296300	5.109	1.510	1.492	1.895	1.604	2.733	2.705	2.532	2.623	1.741	3.218	0.981	2.363	1.722	0.694
TraesCS1B02G269100	0.000	0.060	0.228	0.172	1.384	0.955	0.873	2.056	2.682	3.263	0.000	0.000	1.210	2.055	3.612
TraesCS1B02G272100	0.000	0.887	0.306	0.463	0.306	0.544	0.986	0.453	0.447	0.343	0.000	0.993	0.699	0.199	1.555
TraesCS1B02G272900	0.549	5.292	0.000	0.204	0.115	0.117	0.000	5.307	5.379	4.681	0.248	5.825	0.000	5.432	0.000
TraesCS1B02G269400	0.000	0.840	2.418	2.731	7.133	6.878	5.239	7.025	6.340	6.886	1.624	3.580	6.492	6.619	5.519

TraesCS1B02 G272000	0.000	0.319	0.000	0.056	0.631	1.875	2.299	0.695	0.444	0.098	0.166	0.491	0.252	0.000	0.765
TraesCS2A02 G144900	0.000	0.000	0.000	0.000	5.906	1.891	3.393	0.311	1.788	1.596	3.794	0.000	0.489	0.000	0.000
TraesCS2A02 G145000	0.000	0.000	0.000	0.000	0.055	0.114	0.000	0.138	0.034	0.117	0.000	0.000	0.000	0.000	0.000
TraesCS2B02 G409800	0.000	0.310	0.000	0.000	0.088	0.221	0.693	0.113	0.314	0.315	0.396	0.354	0.000	0.104	0.000
TraesCS2B02 G410400	0.000	0.200	0.000	0.000	0.000	0.113	0.000	0.070	0.017	0.000	0.000	0.036	3.746	0.243	1.564
TraesCS4B02 G259600	1.559	3.835	2.881	4.216	7.170	5.465	5.165	5.517	5.754	5.342	5.196	3.642	6.048	5.078	4.119
TraesCS5A02 G551700	0.000	0.000	0.745	0.000	0.000	0.000	0.000	0.000	0.000	0.000	0.000	0.000	0.000	0.000	0.000
TraesCS7A02 G064700	0.000	0.867	1.441	3.408	3.129	5.682	6.302	3.330	2.457	3.360	3.556	1.011	3.245	3.118	0.000
TraesCS7B02 G432200	1.245	0.000	0.000	0.172	1.077	0.503	0.414	1.178	0.544	0.697	0.838	0.021	0.536	0.828	0.222
TraesCS1A02 G295400	0.000	1.743	0.000	1.281	6.355	5.246	5.502	5.226	5.260	5.721	6.004	0.652	5.634	6.213	7.491
TraesCS1A02 G295500	0.000	0.000	0.000	0.000	0.000	0.000	0.000	0.000	0.000	0.000	0.119	0.262	0.141	0.000	0.000

TraesCS1A02 G295700	0.000	2.123	0.962	0.312	6.588	5.514	5.697	5.554	5.728	6.057	6.114	1.042	5.807	0.775	8.129
TraesCS1A02 G296300	5.109	1.510	1.492	1.895	1.604	2.733	2.705	2.532	2.623	1.741	3.218	0.981	2.363	1.722	0.694
TraesCS3A02 G039000	0.000	0.000	0.000	0.000	0.000	0.000	0.000	0.000	0.000	0.000	0.000	0.000	0.000	0.000	0.000
TraesCS4B02 G259600	1.559	3.835	2.881	4.216	7.170	5.465	5.165	5.517	5.754	5.342	5.196	3.642	6.048	5.078	4.119
TraesCS4B02 G260200	0.547	1.716	3.843	2.669	1.547	2.922	3.547	2.680	2.583	2.277	2.526	1.605	0.853	1.334	0.996
TraesCS5A02 G551700	0.000	0.000	0.745	0.000	0.000	0.000	0.000	0.000	0.000	0.000	0.000	0.000	0.000	0.000	0.000

Supplementary Table S3: Expression data of selected candidate genes under 1 and 6 hours of drought stress.

Genes	1 hour of drought	6 hour of drought
TraesCS1A02G295000	0.000	0.000
TraesCS1A02G295400	1.774	1.230
TraesCS1A02G296200	1.231	1.653
TraesCS1A02G296300	2.449	0.794
TraesCS1B02G269100	0.025	0.053
TraesCS1B02G272100	1.017	0.536
TraesCS1B02G272900	5.393	3.864
TraesCS1B02G269400	3.559	2.205
TraesCS1B02G272000	0.081	0.092
TraesCS2A02G144900	0.000	0.000
TraesCS2A02G145000	0.000	0.000
TraesCS2B02G409800	0.221	0.000
TraesCS2B02G410400	0.030	0.032
TraesCS4B02G259600	3.729	3.640
TraesCS5A02G551700	0.000	0.000
TraesCS7A02G064700	2.020	1.011
TraesCS7B02G432200	0.671	0.352

TraesCS1A02G295400	1.774	1.230
TraesCS1A02G295500	0.000	0.000
TraesCS1A02G295700	2.506	1.045
TraesCS1A02G296300	2.449	0.794
TraesCS3A02G039000	0.000	0.000
TraesCS4B02G259600	3.729	3.640
TraesCS4B02G260200	1.505	1.604
